# Stable Gastric Pentadecapeptide BPC 157 as a Therapy and Safety Key: A Special Beneficial Pleiotropic Effect Controlling and Modulating Angiogenesis and the NO-System

**DOI:** 10.3390/ph18060928

**Published:** 2025-06-19

**Authors:** Predrag Sikiric, Sven Seiwerth, Anita Skrtic, Mario Staresinic, Sanja Strbe, Antonia Vuksic, Suncana Sikiric, Dinko Bekic, Dragan Soldo, Boris Grizelj, Luka Novosel, Lidija Beketic Oreskovic, Ivana Oreskovic, Mirjana Stupnisek, Alenka Boban Blagaic, Ivan Dobric

**Affiliations:** 1 Department of Pharmacology, School of Medicine, University of Zagreb, 10000 Zagreb, Croatiavuksic.antonia55@gmail.com (A.V.); stupnisek@gmail.com (M.S.);; 2 Department of Pathology, School of Medicine, University of Zagreb, 10000 Zagreb, Croatiaskrtic.anita@gmail.com (A.S.);; 3 Department of Surgery, School of Medicine, University of Zagreb, 10000 Zagreb, Croatia; 4 Department of Diagnostic and Interventional Radiology, University Hospital Centre, 10000 Zagreb, Croatia

**Keywords:** BPC 157, angiogenesis, nitric oxide, control/modulation

## Abstract

Although approached through many concepts, the pleiotropic healing issue, specifically, maintaining/reestablishing tissue integrity, remains a central challenge in pharmacology, particularly when the process is misdirected or not properly controlled. Robert and Szabo’s concept of cytoprotection holds that innate cell (epithelial (Robert), endothelial (Szabo)) integrity and protection/maintenance/reestablishment in the stomach is translated to other organ therapy (cytoprotection → organoprotection) via the cytoprotection agent’s effect. Therefore, we defend stable gastric pentadecapeptide BPC 157 therapy’s efficacy and pleiotropic beneficial effects, along with its high safety (LD1 not achieved), against speculation of its negative impact, speculation of angiogenesis toward tumorigenesis, increased NO and eNOS, damaging free radical formation, and neurodegenerative diseases (Parkinson’s disease and Alzheimer’s disease). Contrarily, in wound healing and general healing capabilities, as reviewed, as a cytoprotective agent and native cytoprotection mediator, BPC 157 controls angiogenesis and the NO-system’s healing functions and counteracts the pathological presentation of neurodegenerative diseases in acknowledged animal models (i.e., Parkinson’s disease and Alzheimer’s disease), and it presents prominent anti-tumor potential in vivo and in vitro. BPC 157 resolved cornea transparency maintenance, cornea healing “angiogenic privilege” (vs. angiogenesis/neovascularization/tumorigenesis), and it does not produce corneal neovascularization but rather opposes it. Per Folkman’s concept, it demonstrates an anti-tumor effect in vivo and in vitro. BPC 157 exhibits a distinctive effect on the NO-level (increase vs. decrease), always combined with the counteraction of free radical formation, and, in mice and rats, BPC 157 therapy counteracts Parkinson’s disease-like and Alzheimer’s disease-like disturbances. Thus, BPC 157 therapy means targeting angiogenesis and NO’s cytotoxic and damaging actions but maintaining, promoting, or recovering their essential protective functions.

## 1. Introduction

Commonly, the pleiotropic issue of healing (i.e., specifically, maintaining/reestablishing tissue integrity) remains a central challenge in pharmacology, particularly when the process is misdirected or not properly controlled [1]. Therefore, this review, summarizing the issue already largely reviewed [1,2,3,4,5,6,7,8,9,10,11,12,13,14,15,16,17,18,19,20,21,22,23,24,25,26,27,28,29,30,31,32,33,34,35,36,37,38,39,40], highlights key advantages of BPC 157’s (Body Protection Compound, GEPPPGKPADDAGLV, M.W. 1419, partial sequence of human gastric juice protein BPC, also known as BPC-157, BPC157, BPC 15, PL-10, PL-14736, and bepecin) beneficial pleiotropic effects. The focus was on the particular effects occurring throughout BPC 157’s healing course, which, in general, should be specially considered in terms of providing the outcome that might follow. Implicated specifically were increased angiogenesis, elevated vascular endothelial growth factor (VEGF) levels, upregulation of the early growth response (egr-1) gene, enhanced nitric oxide (NO) and NO-synthase (eNOS) stimulation, and counteraction of increased free radical formation [1]. On the other hand, at the general level, properly accommodated in the healing process, all of these effects can be an essential key to resolving the pleiotropic issue of healing. As a network of tightly interconnected evidence, this can provide a consistent background for the obtained pleiotropic beneficial effects [3,4,5,6,7,8,9,10,11,12,13,14,16,17,18,19,20,21,22,23,24,27,28,29,30,31,32,34,37,39,40]. Therefore, this review highlights that rather than potential concerns [1], these factors serve as a strong beneficial impetus for stable gastric pentadecapeptide BPC 157 therapy [2,3,4,5,6,7,8,9,10,11,12,13,14,15,16,17,18,19,20,21,22,23,24,25,26,27,28,29,30,31,32,33,34,35,36,37,38,39,40].

As mentioned, many reviews on stable gastric pentadecapeptide BPC 157 [1,2,3,4,5,6,7,8,9,10,11,12,13,14,15,16,17,18,19,20,21,22,23,24,25,26,27,28,29,30,31,32,33,34,35,36,37,38,39,40] have been presented by our group [3,4,5,6,7,8,9,10,11,12,13,14,16,17,18,19,20,21,22,23,24,27,28,29,30,31,32,34,37,39,40] and other groups [1,2,15,25,26,33,35,36,38] (Table 1).

These reviews [1,2,3,4,5,6,7,8,9,10,11,12,13,14,15,16,17,18,19,20,21,22,23,24,25,26,27,28,29,30,31,32,33,34,35,36,37,38,39,40] illustrate BPC 157’s largely beneficial activity and its broad therapeutic potential. All of these studies highlight BPC 157’s beneficial effects through a particular innate cytoprotective defensive healing system. In general, the long-standing cytoprotection concept identifies innate epithelial and endothelial cell protection, which was long ago postulated in the stomach [41,42,43,44,45,46,47]. Such protection (cytoprotection) can be extended to other organ therapy via cytoprotective agent application (cytoprotection → organoprotection) [48,49]. It is promptly activated by BPC 157 therapy application [3,4,5,6,7,8,9,10,11,12,13,14,16,17,18,19,20,21,22,23,24,27,28,29,30,31,32,34,37,39,40] and, thereby, its pleiotropic beneficial effects. Notably, both angiogenesis [50,51] and the NO-system [52,53,54,55,56,57] have been fundamental for healing since quite early in the cytoprotection concept. Likewise, a considerable interaction with BPC 157’s beneficial effect is also postulated [11,29]. Also, given that the cytoprotection concept was initiated in the stomach [41], BPC 157 as a stable gastric pentadecapeptide is inherently linked to the cytoprotection concept and this therapeutic paradigm. It is capable of exerting activities of a putative cytoprotection mediator when native and stable in human gastric juice for more than 24 h [3,4,5,6,7,8,9,10,11,12,13,14,16,17,18,19,20,21,22,23,24,27,28,29,30,31,32,34,37,39,40] in contrast to conventional peptidergic growth factors. Illustratively, epidermal growth factor (h-EGF) and transforming growth factor (h-TGF alpha), while stable in water, are rapidly degraded within minutes in gastric juice [58]. BPC 157 has particular stability and is always applied alone, without a carrier, for systemic, peroral, or local application [3,4,5,6,7,8,9,10,11,12,13,14,16,17,18,19,20,21,22,23,24,27,28,29,30,31,32,34,37,39,40]. Unlike standard cytoprotective agents that exhibit only prophylactic effectiveness (shared limitation of activity) [41,42,43,44,45,46,47,48], BPC 157 represents a prototype of a more effective class of cytoprotective agents with both prophylactic and therapeutic ability [3,4,5,6,7,8,9,10,11,12,13,14,16,17,18,19,20,21,22,23,24,27,28,29,30,31,32,34,37,39,40].

Therefore, since the early 1990s, we have presented stable gastric pentadecapeptide BPC 157, and its pleiotropic beneficial effects, acting also via the per oral route [3,4,5,6,7,8,9,10,11,12,13,14,16,17,18,19,20,21,22,23,24,27,28,29,30,31,32,34,37,39,40], as a late advantage of Robert and Szabo’s concept of cytoprotection [41,42,43,44,45,46,47,48,49]. Notably, in the cytoprotection concept, some particular points merit additional emphasis. The introduction of the cytoprotection concept a decade before produced a considerable breakthrough in gastroenterology, first in the prostaglandin issue and then in general. As a principle, the concept established the rat model of the direct cell lesion (necrosis) and the direct defensive immediate response (cytoprotection) [41,42,43,44,45,46,47,48,49]. To reestablish normal circumstances against diverse noxious events, these were stomach lesions induced through intragastric administration of strong alcohol and other necrotizing agents [41,42,43,44,45,46,47,48,49]. In a more complex way, even before epithelial lesions, there were antecedent endothelial injury, thrombi, and stasis and thus Virchow triad circumstances (although not specifically claimed) that needed to be resolved [44,45,46,47]. In addition to continuously maintaining integrity, as a pertinent physiological mechanism, the concept of adaptive cytoprotection was introduced [43]. There were rat stomach lesions in which quick exposure to mild irritants promptly conferred protection against subsequent injury from more severe irritants [43]. Conceptually, this mirrors Selye’s earlier stress theory [59,60]. Seley’s stress concept [59,60] holds that small stress protects against strong stress (general adaptation) [59,60,61]. Overall protection (homeostasis reestablished) (i.e., organoprotection) has long been recognized as the theoretical endpoint of Selye’s stress response [59,60,61,62]. Likewise, a pleiotropic beneficial effect (i.e., organoprotection) is the theoretical outcome of the cytoprotection concept and cytoprotection agents’ application [41,42,43,44,45,46,47,48,49]. Also, BPC 157 counteraction includes damages induced by both cyclooxygenase *(COX)-1* and *COX-2* blockers [30] and markedly enhanced adaptive cytoprotection [63,64]. Furthermore, in addition to counteracting the primary intragastric alcohol-induced stomach lesions, BPC 157 counteracted all other lesions, including those of the brain, heart, lung, liver, and kidney, as well as thrombosis and vascular failure, peripherally and centrally, intracranial, portal, and caval hypertension, and aortal hypotension. There was the reversal of occlusion/occlusion-like syndrome as a whole [4,10] and the reversal of advanced Virchow triad circumstances. The novel point was the rapid activation of the collateral rescuing pathways (i.e., activation of the azygos vein’s direct blood flow delivery) to reestablish the reorganized blood flow [4,10].

Thus, the pleiotropic beneficial effect of BPC 157 therapy entirely follows the principle of the concept of cytoprotection. The cytoprotection concept holds innate cell (epithelial (Robert) [41,42,43], endothelial (Szabo) [44,45,46,47]) integrity, even against opposite damaging necrotizing agents (i.e., strong alcohol, boiling water, strong acid, strong base [41]) and protection/maintenance/reestablishment in the stomach to be translated to other organ therapy (cytoprotection → organoprotection) [48,49] via the cytoprotection agent’s effect. This implicates a regular corresponding effect on angiogenesis and NO-system functioning even in opposite conditions [3,4,5,6,7,8,9,10,11,12,13,14,16,17,18,19,20,21,22,23,24,27,28,29,30,31,32,34,37,39,40]. Likewise, this implicates high effectiveness (i.e., BPC 157 therapy is effective in the 10 µg–10 ng/kg range). In toxicology studies, BPC 157 exhibited a negative limit test, 2 g/kg i.v. or i.g., without adverse effects in mice, and a lethal dose (LD1) was not achieved [3,4,5,6,7,8,9,10,11,12,13,14,16,17,18,19,20,21,22,23,24,27,28,29,30,31,32,34,37,39,40]. Later, it was effectively used in ulcerative colitis trials (phase II) without adverse effects [63,64].

As we reviewed [21] and mentioned before, stable gastric pentadecapeptide BPC 157 is quite distinctive from standard angiogenic peptides in many aspects. As it is native and stable in human gastric juice for more than 24 h, as a cytoprotection mediator always given alone, it exerts (according to the original cytoprotection concept) its beneficial effects pleiotropically. This can be advantageous over standard angiogenic peptides as no carrier is used; all of these effects are unmistakably attributed. Contrarily, standard angiogenic peptides often need various carrier additions (and therefore peptide+carrier(s) and an undefined effect). These might make BPC 157’s results even more reliable, given that successfully using different application routes (including per oral) in one model established congruent efficacy in whatever application route [3,4,5,6,7,8,9,10,11,12,13,14,16,17,18,19,20,21,22,23,24,27,28,29,30,31,32,34,37,39,40]. Namely, as pointed out with alcohol lesions but also with other occlusion/occlusion-like syndromes, with BPC 157 therapy, there is a distinctive beneficial action [4,10]. Pleiotropic beneficial effects occurred in the lesions in the brain, heart, lung, liver, kidney, and gastrointestinal tract, as well as in the reversal of severe multiorgan and vessel failure, reversal of occlusion/occlusion-like syndrome as a whole [4,10], and reversal of advanced Virchow triad circumstances. These occurred without causing harm to other organs [4,10]. This sided with the mentioned safety evidence, as BPC 157 exhibited a general lack of toxicity. A negative limit test, 2 g/kg i.v. or i.g., without adverse effects was shown in mice, and a lethal dose (LD1) was not achieved, which also shows metabolites without harmful effect [3,4,5,6,7,8,9,10,11,12,13,14,16,17,18,19,20,21,22,23,24,27,28,29,30,31,32,34,37,39,40].

This might be important, as the stable gastric pentadecapeptide BPC 157 has been a special topic [1,2,3,4,5,6,7,8,9,10,11,12,13,14,15,16,17,18,19,20,21,22,23,24,25,26,27,28,29,30,31,32,33,34,35,36,37,38,39,40]. Its pleiotropic beneficial effects, significance as a possible cytoprotection mediator, neurotransmitter, eye therapy, tendon, muscle (striated, smooth, and heart muscle), junction (neuromuscular, osteotendinous, myotendinous, muscle-to-bone attachment), angiogenesis, and NO-system functions, and its considerable role in brain–gut axis and gut–brain axis functioning were presented in several reviews given by our group [3,4,5,6,7,8,9,10,11,12,13,14,16,17,18,19,20,21,22,23,24,27,28,29,30,31,32,34,37,39,40] and other groups [1,2,15,25,26,33,35,36,38], as well. As a highlight, BPC 157 as a neurotransmitter (or as neurotransmitter-like) can offer a network of interconnected evidence [6,9] previously envisaged in the implementation of cytoprotection effects (i.e., a cytoprotection mediator holds a response specifically related to preventing or recovering damage as such [41]). Although BPC 157 lacks general standard neurotransmitter criteria, in classic terms, particular evidence of a consistent beneficial effect shows that BPC 157 therapy counteracts dopamine, serotonin, glutamate, GABA, adrenalin/noradrenalin, acetylcholine, and NO-system disturbances. This occurs regardless of the effect specifically related to their receptors, including blockade, over-activity, destruction, depletion, tolerance, sensitization, and channel disturbance counteraction [6,9].

Such an innate cytoprotective defensive healing system will correspondingly establish the consistent beneficial effects of stable gastric pentadecapeptide BPC 157 therapy on angiogenesis and the NO-system [3,4,5,6,7,8,9,10,11,12,13,14,16,17,18,19,20,21,22,23,24,27,28,29,30,31,32,34,37,39,40]. As mentioned, the specific issue could be a strong effect on increasing angiogenesis, increased VEGF, increased egr-1 gene, increased NO, or eNOS stimulation and counteraction of increased free radical formation. From a negative perspective [1], outside of the cytoprotection concept, or if cytoprotection is not a valid concept, these might indicate the uncertain complexity and multifaceted nature of the biological activity and its interaction with multiple systems. Thus, there could be unescapable risks of unanticipated adverse effects due to pleiotropic effects [1]. These events could combine a threatening chain of events until the final harm [1]: ↑angiogenesis → carcinoma, ↑VEGF → carcinoma; ↑egr-1 gene → carcinoma; ↑NO → ↑free radicals → neurodegenerative disease (Parkinson’s disease, Alzheimer’s disease).

Alternatively, all of these items, properly accommodated by therapy, might be a resolving key to beneficial action [3,4,5,6,7,8,9,10,11,12,13,14,16,17,18,19,20,21,22,23,24,27,28,29,30,31,32,34,37,39,40]. This might be the updated cytoprotection concept [3,4,5,6,7,8,9,10,11,12,13,14,16,17,18,19,20,21,22,23,24,27,28,29,30,31,32,34,37,39,40], as innate epithelial and endothelial cell protection was long ago postulated in the stomach [41,42,43,44,45,46,47,48]. This might be targeting angiogenesis and NO’s cytotoxic and damaging actions but maintaining, promoting, or recovering their essential protective functions [3,4,5,6,7,8,9,10,11,12,13,14,16,17,18,19,20,21,22,23,24,27,28,29,30,31,32,34,37,39,40]. This would occur as a highly controlling beneficial action, such as activation depending on the disturbed circumstances, an effect ascribed to the BPC 157 therapy effect.

Conceptually, this should be a large network of interconnected forms of beneficial evidence supporting each other and fully supporting these particular relations and the essential controlling role of pentadecapeptide BPC 157 in angiogenesis and NO-system function.

## 2. Angiogenesis

Angiogenesis is the physiological process through which new blood vessels form from pre-existing vessels. Angiogenesis as a normal physiological function has an important role in the process of healing.

As it is widely acknowledged, the pioneering work of Judah Folkman defined the concept of angiogenesis. A particular highlight was the induction of corneal neovascularization and angiogenesis that can lead to tumorigenesis [65,66,67,68,69,70,71,72,73].

In principle, BPC 157 therapy has demonstrated a large range of beneficial effects in different forms of tissue healing [3,4,5,6,7,8,9,10,11,12,13,14,16,17,18,19,20,21,22,23,24,27,28,29,30,31,32,34,37,39,40]. As such, this suggests the realization of a particular implementation of angiogenesis. Likewise, there are the distinctive needs for angiogenesis in different tissues (cornea vs. other tissues [65,66,67,68,69,70,71,72,73]). Therefore, the large healing effect shared in many tissues implies specific control amid precise regulatory mechanisms. This can ensure the optimal realization of the healing process [3,4,5,6,7,8,9,10,11,12,13,14,16,17,18,19,20,21,22,23,24,27,28,29,30,31,32,34,37,39,40].

Otherwise, if increased angiogenesis appears as dysregulation, as a likely possibility, regardless of beneficial effects, there is a considerable probability that increased angiogenesis precedes the complications and impedes uncontrolled VEGF, the egr-1 gene, carcinoma, and the NO-system [1].

In pursuing cytoprotection research, a strong angiogenic effect of BPC 157 has been established, along with resolving external and internal wound healing [3,4,5,6,7,8,9,10,11,12,13,14,16,17,18,19,20,21,22,23,24,27,28,29,30,31,32,34,37,39,40]. The noted angiogenic effect markedly exceeds standard antiulcer agents [74]. The method used in these first studies was similar to that used by Szabo’s group [75]. In 3 or 7 days, the implantation of two sponges on the back of rats led to increased formation of new vessels along with granulation tissue formation [74]. Further research has revealed a network of interrelated beneficial forms of healing from BPC 157 therapy exhibiting distinctive effects on angiogenesis (i.e., cornea [76,77,78,79] vs. tendon, muscle, and other tissue healing [80,81,82,83,84,85,86,87,88,89,90,91]) (for a review, see, in particular [8,16]). Underscoring a distinct therapeutic relationship between BPC 157, this appears as a distinctive argument for BPC 157/angiogenesis’s specific healing relationship and tissue-specific angiogenesis [8,16]. These beneficial effects are complementary to the reported beneficial effects specifically noted with VEGF [84,92,93,94,95,96], NO-system [11,29,92,93,94,97,98,99], egr-1 gene [100], and carcinoma [22,95] models.

An instructive illustration is a study by Hsieh and collaborators [94]. The evidence combined BPC 157 with the time-dependently activated VEGFR2-Akt-eNOS signaling pathway, the increase in endothelial tube formation (chick chorioallantoic membrane (CAM)) (both suppressed by Dynasore, an endocytosis inhibitor), the increased expression and internalization of VEGFR2, and essential acceleration of blood flow recovery and vessel number to rescue hind limb ischemia in rats with a completely excised femoral artery [94].

Thus, unlike angiogenic peptides [65,66,67,68,69,70,71,72,73], it is evident that BPC 157 angiogenesis [3,4,5,6,7,8,9,10,11,12,13,14,16,17,18,19,20,21,22,23,24,27,28,29,30,31,32,34,37,39,40] does not align with Folkman’s first essential angiogenesis rule [76]. Unlike angiogenesis, BPC 157 does not share mechanisms and pathways through which an agent could induce angiogenesis, which can be harmful. First, BPC 157 therapy induces the healing of corneal ulcers [76]. This goes with maintaining corneal transparency, opposing corneal neovascularization, and resolving cornea “angiogenic privilege” [76]. The therapeutic effect in eye pharmacology is extended to the counteraction of glaucoma and retinal ischemia [8]. Likewise, it induces beneficial effects on other tissues (i.e., advanced healing of tendons and muscle, recovery of liver cirrhosis, and counteraction of portal hypertension) (see Section 2.1, Section 2.2 and Section 2.3). Second, on the other hand, BPC 157 therapy [76] implements the other part of Folkman’s cornea concept. Corneal neovascularization goes with tumor growth, inhibited corneal neovascularization, and inhibited tumor growth [65,66,67,68,69,70,71,72,73]. Because it inhibits both processes, it exhibits particular anti-tumor potential [22,95] (see Section 2.4 and Section 2.5).

### 2.1. Angiogenesis, Cornea Neovascularization, and Tumor Growth

In 1971, Judah Folkman published his theory of angiogenesis [70]. New vessels appeared as the essential key to tumor growth [71]. Therefore, there is the existence of a family of angiogenic peptides [71], and the removal of an angiogenic stimulus leads to the regression of neovascularization and tumor growth [72]. But, the essential point is Folkman and colleagues’ neovascularization cornea evidence that macromolecules and proteins can be released from polymers implanted in the cornea. This methodology (cornea neovascularization) was critical for proof of such angiogenic bioactivity of a given molecule in vivo [66,73].

Contrarily, with BPC 157 eye administration, there were no signs of eye irritation or neovascularization in albino rabbits, even with 10 mg/mL of carbopol gel (Acute eye irritation/corrosion study, TNO Pharma, 8 July 2004). Moreover, as a part of its cytoprotective activity (healing specific to a given organ), confronted with a corneal ulcer that could not heal to any extent in rats, BPC 157 therapy cured severe corneal lesions and maintained corneal transparency [76]. After injury induction, BPC 157 therapy successfully closed perforating corneal incisions in rats and rapidly restored corneal transparency. This effect is quite consistent given the regimens used, i.e., 2 pg/mL, 2 ng/mL, and 2 µg/mL distilled water, two eye drops/left rat eye immediately after injury induction, and then every 8 h up to 120 h. All controls developed new vessels that grew from the limbus to the penetrated area and had no transparency. Contrarily, BPC-157-treated rats generally had no new vessels, and those that did form in the limbus did not make contact with the penetrated area. As an illustration of the full effectiveness against otherwise incurable lesions, BPC 157 significantly accelerated the healing process in all 2 µg and 2 ng BPC 157 treated eyes, starting 24 h after the injury, and the fluorescein and Seidel tests became negative. The epithelial defects completely healed at 72 h (2 µg BPC 157 treated group) and at 96 h (2 ng BPC 157 treated group) after injury. Aqueous cells were absent at 96 h and 120 h after injury in the 2 µg and 2 ng BPC 157 treated groups, respectively. Note that these have implemented as a crucial diagnostic tool in ophthalmology. The Seidel test detects aqueous humor leakage and corneal perforations or a significant injury and assesses the integrity of the eye’s anterior segment. The fluorescein test detects abnormalities on the surface of the eye, including corneal abrasions, foreign bodies, and other pathologies. Thus, BPC 157 eye drops successfully close perforating corneal incisions in rats [76].

Therefore, there is consistent evidence that BPC 157 did not produce, but opposed, essential corneal neovascularization, healed corneal ulcers, and maintained transparency [76]. This means that it has strong distinctions from angiogenic peptides, i.e., fibroblast growth factor (FGF), EGF, and VEGF, known to produce neovascularization in the cornea [71,101,102,103]. Therefore, BPC 157 does not share their potentially threatening angiogenesis chain of events (i.e., carcinoma, NO-system) [104,105,106,107,108,109].

Likewise, after total debridement of the corneal epithelium and a completely denudated cornea, BPC 157 accelerated corneal recovery and maintained corneal transparency [77] (i.e., 2 pg/mL, 2 ng/mL, and 2 µg/mL distilled water, two eye drops/left rat eye immediately after injury induction, and then every 8 h up to 120 h). After lacrimal gland extirpation, BPC 157 counteracted the damaging effects of dry eye syndrome in rats [78,79]. Recovered corneal injuries and recovered corneal transparency were exemplified in rats who underwent complete corneal abrasion [77], corneal ulceration [76], lacrimal gland-removal-induced dry eye, or corneal insensitivity [78,79]. Thus, corneal neovascularization was strongly counteracted, whatever the cause. Such particular vascular function recovery in corneal injuries (i.e., maintained transparency after complete corneal abrasion or corneal ulceration and counteracted dry eye after lacrimal gland removal or corneal insensitivity) [76,77,78,79] is also implicated in other beneficial effects of BPC 157 eye therapy (for a review, see, in particular [8]). These beneficial effects were shown in the counteraction of glaucoma (i.e., normalization of increased intraocular pressure, maintained retinal integrity, recovered pupil function), the counteraction of retinal ischemia, and the control of pupil function [110,111,112,113].

### 2.2. Corneal Transparency to Illustrate the Consistent Organ-Specific Healing Angiogenesis Effect

**Figure 1:**
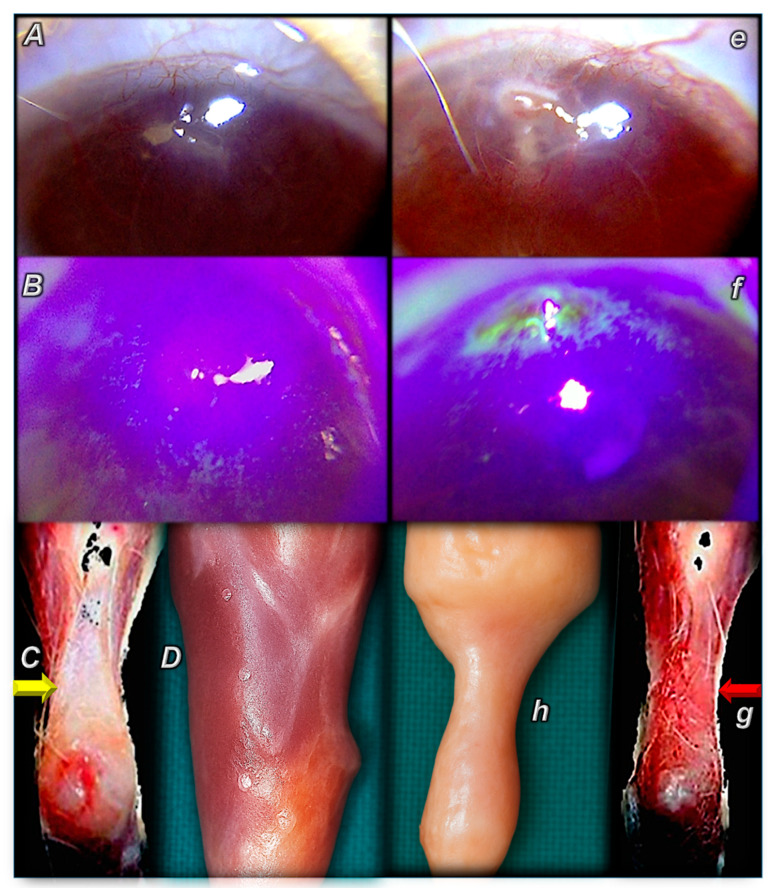
Illustrative distinctive presentation of control (small letters) and BPC 157-treated rats (capital letters). Summary of the effects of controlled angiogenesis, opposed angiogenesis (cornea) [76], advanced angiogenesis (tendon, muscle) [88,89] (BPC 157 therapy) (**A**–**D**) vs. regularly failed angiogenesis (control), (**e**–**h**) corneal neovascularization, failed healing tendon and muscle. *BPC 157 therapy*. (**A**,**B**) Corneal ulcer (60× magnification). BPC 157 2 pg/mL, 2 ng/mL, and 2 µg/mL distilled water, two eye drops/left rat eye immediately after injury induction, and then every 8 h up to 120 h. (**A**) Absence of edema in the site of ulceration, new vessels markedly attenuated, corneal transparency, no signs of inflammatory process, on post-operative day 3. (**B**) On post-operative day 5, staining the cornea with fluorescein dye and examination under blue light shows a negative fluorescein test, epithelial defect at the site of ulceration healed, and the defect does not persist. The cornea is flat, and there are no abnormalities. (**C**,**D**) Gross presentation after the rat’s Achilles tendon was sharply transected from the calcaneal bone (**C**) and after major muscle transection (quadriceps muscle) (**D**) (yellow arrow). BPC 157 regimens 10 µg, 10 ng, 10 pg/kg, intraperitoneally, once daily. (**C**) Detached tendon, no defect between the tendon stump and the calcaneal bone at post-operative day 10. The edge of the tendon stump cannot be recognized (osteotendon junction re-established). (**D**) Improved gross presentation of transected muscle, muscle presentation with regeneration and absent marked atrophy, at day post-surgery day 72, and there was always functional, biomechanical, microscopical, and immunohistochemistry healing improvement. *Controls*. (**e**,**f**) Corneal ulcer (60× magnification). (**e**) Edema at the site of ulceration, growth of new vessels, corneal opacity, and poor transparency on post-operative day 3. The inflammatory process is active. (**f**) At postoperative day 5, staining the cornea with fluorescein dye and examination under blue light shows a positive fluorescein test, epithelial defects (green areas) at the site of ulceration, the surface of the cornea is not flat, and defects and inflammatory conditions still persist. (**g**,**h**) (red arrow) Gross presentation after the rat’s Achilles tendon was sharply transected from the calcaneal bone (**g**) and after major muscle transection (quadriceps muscle) (**h**). (**g**) Significant gap between the tendon edge and the bone, with a clear stump at postoperative day 10. (**h**) Largely atrophied muscle on post-surgery day 72.

Finally, the rapid regaining of corneal transparency realized by BPC 157 therapy (cornea vs. other organs), a particular distinctive huge wound healing capacity, was specifically reviewed [16]. This implies that healing is realized depending on the organ involved; angiogenesis is opposed (cornea) [76,77,78,79], or a strong angiogenic healing effect is consistently noted [80,81,82,83,84,85,86,87,88,89,90,91] (Figure 1, Table 2). Illustratively, there was healing of the transected tendon [84,90], the transected ligament [82], crushed [83,84,87], denervated [85], or transected [84,85] muscle, the osteotendinous junction following Achilles’ tendon detachment [86,89,91] and the myotendinous junction [80], and muscle-to-bone reattachment [81]. Note that mature tendons have hypocellular, hypovascular, and hyponeural structures [114,115,116], while they are during development rich in cells and metabolically active and contain a high number of blood vessels [117]. This can illustrate regularly present specificity in BPC 157 angiogenesis. Unlike a damaged cornea [76,77,78,79], in the healing of a transected Achilles tendon already at postoperative day 4, BPC 157 treated rats have large fields of dense mature collagen, illustrating consistent organ-specific healing effect cellularity and well-formed capillaries and small vessels [90], while control rats exhibit only some young capillaries.

**Figure 2:**
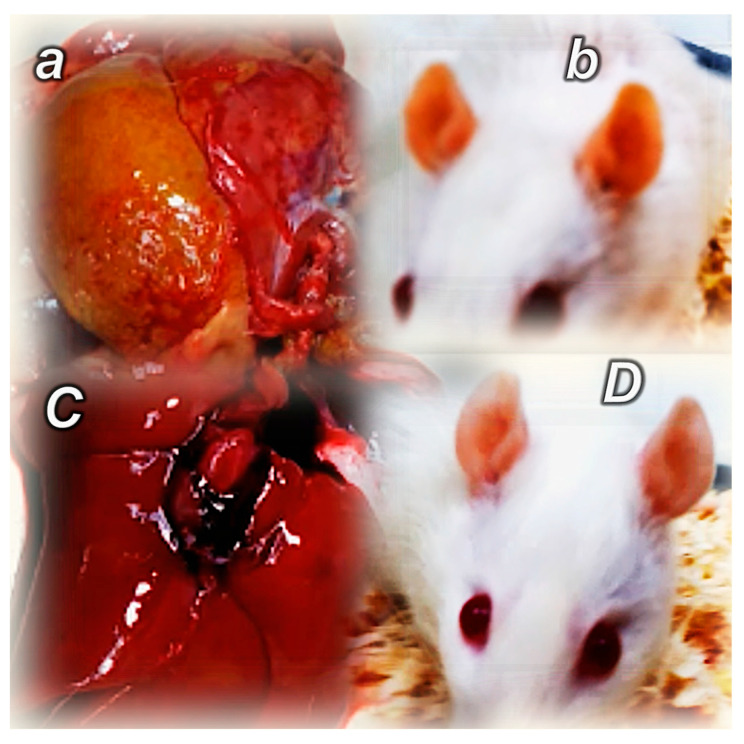
Illustrative distinctive presentation of control (small letters) and BPC 157-treated rats (capital letters). Summary of the effects of controlled angiogenesis, pathologic angiogenesis (bile duct ligation-induced liver cirrhosis, 8 weeks) (control) (**a**,**b**) [118], opposed pathologic angiogenesis (BPC 157 therapy) (**C**,**D**). Gross yellow presentation of the liver and yellow ear presentation (control) (**a**,**b**), presentation of the liver and ear close to normal presentation (BPC 157 therapy) (**C**,**D**).

Another instructive BPC 157/angiogenesis example can be the beneficial effect of BPC 157 therapy on liver lesions, especially cirrhosis [118]. The angiogenesis and disruption of liver vascular architecture have been linked to progression to cirrhosis and liver cancer in chronic liver diseases, which contributes to both increased hepatic vascular resistance and portal hypertension and decreased hepatocyte perfusion [119,120,121]. Pathologic angiogenesis and hypoxia synergistically disrupt normal tissue repair, thereby promoting the development of liver fibrosis [119,120,121]. Contrarily, in bile duct occluded rats, in an 8-week study [118], BPC 157 was given continuously (intraperitoneally once a day or perorally (continuously in drinking water)) or only once as a direct bath application. Liver weight was not increased, and ascites was eliminated. Microscopy presentation documented the smaller intensity of architectural changes (fibrosis and cirrhosis); lower necroinflammatory score; smaller alpha-smooth muscle actin (α-SMA) distribution; and smaller Ki-67 distribution. Smaller were serum enzymes and bilirubin values. Normalized were malondialdehyde (MDA)- and NO-levels in the liver, next to Western blot of NOS2 and NOS3 in the liver tissue, and decreased IL-6, TNF-α, and IL-1β levels in liver tissues. Annihilation of portal hypertension consistently occurred. Despite bile duct ligation, portal pressure did not develop. With the late application of BPC 157 therapy in bile duct ligated rats with already advanced liver cirrhosis, portal hypertension disappeared and did not reappear. Note, however, that the particular points (i.e., hepatic endothelial dysfunction or remodeling and constriction of the intrahepatic sinusoidal vasculature [119,120,121]) were not studied with BPC 157 therapy. Still, it is safe to conclude that a vicious circle between liver fibrosis, cirrhosis, portal hypertension, and pathologic angiogenesis did not occur, or, once it had already advanced, there was a rapid reversal [118] (Figure 2, Table 2).

Consequently, BPC 157 therapy of corneal ulcers results in corneal transparency close to that of healthy avascular cornea [76]. This highlights the resolved “corneal angiogenic privilege”, a corneal avascular state known to be essential for cornea healing and transparency maintenance [122,123,124,125,126,127,128]. Moreover, as an additional particular beneficial point in eye therapy [8,16], with BPC 157 therapy, resolving a corneal ulcer occurs alongside beneficial effects in rat glaucoma [113] and retinal ischemia [112]. Moreover, “corneal angiogenic privilege” and corneal avascular state are commonly understood as a critical and sensitive balance between anti-angiogenic and pro-angiogenic mechanisms and antiangiogenic factors vs. proangiogenic factors (i.e., upregulated after wound healing even in the absence of new vessels) (for a review, see, i.e., [126,127,128]). Thus, corneal ulcer healing (opposed neovascularization, maintained transparency) [76] accords with the beneficial promoting effects (angiogenesis) in other tissues (i.e., tendon, muscle) [80,81,82,83,84,85,86,87,88,89,90,91] and beneficial counteraction (pathologic angiogenesis) in other tissues (i.e., liver) [118]. This can be taken as a piece of compelling evidence that BPC 157 (i.e., advanced healing) affects and controls a balance between competing proangiogenic and antiangiogenic mediators [8,16] (Figure 1, Figure 2). As an example of BPC 157’s impact, there is control of the proangiogenic VEGF (although the specific effect of BPC 157 therapy on other mediators has not been studied so far) (see Section 2.3).

Furthermore, long before angiogenesis begins, BPC 157 therapy rapidly activates collateral blood vessels [4,10]. These occurred in reversing thrombosis and Virchow triad circumstances in counteraction of the severe multiorgan and vascular failure of occlusion/occlusion-like syndromes [4,10]. Illustratively, for counteraction, there was azygos vein direct blood flow delivery, providing the reestablishment of the reorganized blood flow, and full rescue was achieved [129,130,131,132,133,134,135,136,137,138,139,140,141,142]. Note, portal hypertension, along with caval and intracranial hypertension and aortal hypotension, was regularly eliminated/attenuated [129,130,131,132,133,134,135,136,137,138,139,140,141,142]. Furthermore, Fourier transform infrared spectroscopy evidenced in the vessel wall, within minutes, a rapid change in the lipid contents and protein secondary structure conformation, produced instantly via BPC 157 therapy [143]. This shows support for the vessel’s function even in the worst circumstances.

### 2.3. BPC 157 and VEGF

Illustrating such control of angiogenesis, this could also be performed with VEGF. Indicatively, in damaged muscle and tendon, advanced healing through BPC 157 therapy means a particular course of VEGF expression (immunohistochemistry, along with CD34 and FVIII presentation). It increased in the first days, while subsequently decreasing in later days. Thus, it successfully reflected healing already at earlier points [84]. In general, this is fully compatible with the healing of tendon and muscle injuries that could not spontaneously heal, such as a transected or detached tendon, transected, crushed, and denervated muscle, and severed junction reestablishment (i.e., osteotendinous, myotendinous, and muscle-to-bone attachment) [80,81,82,83,84,85,86,87,88,89,90,91]. Moreover, well-controlled angiogenesis and wound healing [16] through BPC 157 therapy might appear in the simultaneous healing of different tissues. This occurred with consistent healing of various external and internal fistulas [19] and gastrointestinal anastomoses [5].

Likewise, further VEGF-A findings should be regarded as specific support for well-controlled particular healing effects. These should be considered alongside the findings that BPC 157 treatment increased the expression of VEGF-A in alkali-burn skin wounds in rats [96]. As mentioned before, these were also angiogenesis-promoted in the CAM assay and the tube formation assay and accelerated the blood flow recovery and vessel number in rats with hind limb ischemia [94]. BPC 157 upregulates VEGFR2 expression in rats with hind limb ischemia and in endothelial cell culture. BPC 157 promotes VEGFR2 internalization in association with VEGFR2-Akt-eNOS activation without the need for other known ligands or shear stress [94]. Likewise, as a healing effect in counteracting the recurrence of acetic acid gastric lesions by clopidogrel, BPC 157 therapy counteracted disturbed angiogenesis [92]. This was along with counteraction of clopidogrel-induced down-regulation of the *VEGF-A* and *VEGFR1*, subsequently inactivated *AKT* signaling pathway, and induced phosphorylation of *p38/MAPK* and *ERK/MAPK* [92].

Finally, the mRNA expression studies showed decreased VEGF gene expression in perforated rat stomachs [144] and strongly elevated VEGFr2 gene expression in the brains of stroked rats that received BPC 157 therapy following reperfusion [145].

### 2.4. BPC 157 and Tumor

VEGF acts as a potent mitogenic growth factor in various cell culture systems [146,147], exerting mitogenic activity by signaling via the Mitogen Activated Protein Kinase (MAPK) pathway in various cancer cells. The blockage of VEGF/MAPK signaling by small kinase inhibitors inhibits cancer growth and spreading [148,149,150,151].

BPC 157 counteracts the VEGF tumor-promoting effect and inhibits cell growth and VEGF signaling via the MAPK kinase pathway in the human melanoma cell line [95]. It was evidenced that Western blot analysis of MAPK signaling pathway showed decreased phosphorylation of ERK (pERK/MEK) in cells treated with 10 ng of BPC 157 and in cells treated with the combination of 10 ng/mL of VEGF and 10 ng/mL of BPC 157 in comparison to control cells and VEGF-stimulated cells. Given the striking decrease of 55% of cells entering the S-phase of the cell cycle upon treatment with 2 ng/mL and 10 ng/mL of BPC 157 and decreased ERK phosphorylation, it was suggested that BPC 157 acts as an antimitogenic agent by preventing ERK phosphorylation and the propagation of the mitogenic signal via the MAPK signaling cascade, triggered by VEGF [95].

The evidence that gastric pentadecapeptide BPC 157 acts as a potent kinase inhibitor of VEGF and MAPK signaling and an inhibitor of melanoma cell growth in vitro [95] should be taken along with further evidence. In vivo, in mice, BPC 157 therapy considerably reduced the number of lung metastases induced by melanoma B-16 (personal communication). Moreover, the potential of BPC 157 as a fundamental agent targeting the signaling process implicated in cancer cachexia was also raised. In mice bearing colon carcinoma C26, BPC 157 therapy counteracts severe muscle cachexia and weight loss, improving anabolic pathways while counteracting catabolic pathways and cachexia mediators and, most importantly, prolonging survival time [22].

Therefore, it seems likely that BPC 157 can control VEGF activity (organizing angiogenesis in healing, counteracting tumor-promoting effects).

### 2.5. BPC 157 vs. Procedures and Agents That Particularly Promote Epithelial Growth, a Widely Acknowledged Fear of Inappropriate Levels of Proliferation, or Potentially Carcinomatous Changes

In addition, there were indicative effects of BPC 157 therapy in a prolonged investigation of the recovery of rats after 70% hepatectomy (liver regeneration) [152,153,154], and, with a short bowel [155], constant weight gain and, finally, pre-operative values are achieved. Fully recovered short bowel syndrome provided finally defined physiological adaptive enlargement, adapted-to-normal tissue ratios, muscle thickness with a fourfold increase, villus height and crypt depth with a twofold increase, and, later, a discrepant decrease of the inner (circular) muscular layer, with all intestinal wall layers accordingly adapted [155]. These beneficial effects of BPC 157 therapy [152,153,154,155] contrast with the use of agents that particularly promote epithelial growth, a widely acknowledged fear of inappropriate levels of proliferation, or, potentially carcinomatous changes, either within the gastrointestinal tract or elsewhere [156].

Finally, we can conclude that Folkman’s cornea concept equates cornea neovascularization with tumor growth and that removing an angiogenic stimulus leads to the regression of neovascularization and tumor growth [157]. Thus, inhibiting corneal neovascularization, all other effects of BPC 157 on angiogenesis, and the anti-tumor effects noted could suggest a particular anti-tumor effect that could be consistently achieved through BPC 157 therapy [3,4,5,6,7,8,9,10,11,12,13,14,16,17,18,19,20,21,22,23,24,27,28,29,30,31,32,34,37,39,40].

### 2.6. BPC 157/egr-1

In our view, BPC 157 therapy for severe wound healing in many tissues, as reviewed [16], emphasized an additional similar controlling regulatory point. This stimulated the expression of the egr–1 gene, as it appeared as a combined effect, along with increased expression of its repressor nab2 gene [98]. There can be an immediate solution, along with its modulatory controlling effect described above for VEGF (see above, Section 2.3 and Section 2.4), as the promoter of the egr-1 gene also mediates angiogenesis [158,159] and induces cytokine and growth factor generation and early extracellular matrix (collagen) formation. This effect appeared more rapidly than the recombinant human platelet-derived growth factor homodimer of B-chains (PDGF-BB) [98]. BPC 157 expressing egr–1 mRNA was observed after 15 min, followed by almost immediate expression of nab2 mRNA [100]. Thus, as suggested [100], the negative feedback loop established between egr-1 and nab2 [160] that would likely appear immediately with BPC 157 therapy can be a particular key in the prompt healing effect of BPC 157 therapy [16,100]. Therefore, the evidence of controlling both the egr-1 gene and its repressor nab2 gene through BPC 157 therapy [100] provides the basis for the belief that with BPC 157, uncontrolled egr-1 gene expression would not occur. Furthermore, the beneficial effects of BPC 157 therapy fully involved recovery of the egr-1-gene-implicated pathology (i.e., cardiovascular, liver, and brain) [3,4,5,6,7,8,9,10,11,12,13,14,16,17,18,19,20,21,22,23,24,27,28,29,30,31,32,34,37,39,40]. Enhanced expression of egr-1 was combined with a multitude of pathologies (i.e., atherosclerosis [159], stenosed calcific valvular disease [161], cardiac hypertrophy [162,163], cerebral ischemia [164], and tumor progression [158,165,166,167]). These events, as adverse effects, due to the described effect of stimulated expression of the egr–1 gene along with its repressor nab2 gene [100], when confronted with BPC 157 application, would provide a resolving therapy effect.

### 2.7. BPC 157/NO-System

All of these beneficial effects of BPC 157 therapy can be achieved along with the NO-system (see below), and the controlling/modulating angiogenesis should occur along with similar corresponding controlling/modulating NO-system functioning [11,29,92,93,94,97,98,99].

The evidence that the BPC 157 principle accommodates the NO-system’s function as a whole is based on its close interaction with NO-agents, given that each NO-agent would mimic particular endogenous circumstances. This point was resolved in all of our studies (for a review, see [11,29]) with the simultaneous application of L-NAME, the application of L-arginine, and the application of L-NAME and L-arginine together (L-NAME+L-arginine) (NO-agents’ tripled application). Therefore, there is consistent evidence that BPC 157 counteracted the effects of L-NAME, counteracted the effects of L-arginine, as well, and overwhelmed the effects of L-NAME+L-arginine. Thus, BPC therapy can affect the NO-system as a whole and NO-system inhibition vs. NO-system over-stimulation vs. NO-system immobilization, thus controlling/modulating NO-system functioning. As an illustration, it opposed hypertension and pro-thrombotic effects (L-NAME) [11,29,97,98,99], as well as hypotension and anti-thrombotic (L-arginine) effects [11,29,97,98,99].

Therefore, the consistent beneficial effects of BPC 157 therapy exclude poorly controlled NO increase (and therefore the consequent chain of negative events) (see below, Section 3.2) and poorly controlled effects on the increase of NOS gene expression (NOS-1, NOS-2, and NOS-3 gene expression). With the consistent beneficial effects of BPC 157 therapy, these were either increased or decreased, noted at different time points and depending on the specific support of particular well-controlled healing effects [80,144,145,168,169,170,171].

An interesting example is the healing of duodenocolic fistulas in rats through BPC 157 therapy very early upon the creation of a duodenocolic fistula [169]. BPC 157 therapy rapidly induces vessel “recruitment”, “running” toward the defect. It does so simultaneously at the duodenum and the colon, providing numerous collaterals and branching amid strongly elevated (NOS-2) and decreased aspects (COX-2, VEGF A, NOS-1, NOS-3, nuclear factor-kappa-B-activating protein (Nfkb) gene expression (the mRNA expression studies) [169]. As a way of understanding how BPC 157 may act beneficially in perforated stomach lesions, we demonstrated a timely, likely gene-specific congruence [144]. In the healing of the perforated stomach in the first 15 min, there was initially elevated NOS-2 and decreased VEGFa, then elevated NOS-2 and decreased VEGFa, then elevated COX-2, NOS-1, NOS-2, NOS-3, and then elevated COX-2, NOS-2, and decreased NOS-3 gene expression [144]. Similarly, the healing of spinal cord injured rats through BPC 157 therapy is interesting, as very early, upon injury, there is a decrease of hematoma amid strongly elevated NOS-1, NOS-2, and NOS-3 gene expression (the mRNA expression studies) [170]. Likewise, the healing of isoprenaline–myocardial infarction rats through BPC 157 therapy is interesting, as one day after isoprenaline injury, a decrease of COX-2 and NOS-3 (NOS-2 not affected) gene expression occurred (the mRNA expression studies) [168]. In the reversing reperfusion stroke, after 60 min, mRNA expression studies at 1 and 24 h reperfusion time provided strongly elevated (Egr1, Akt1, Kras, Src, Foxo, Srf, Vegfr2, NOS-3, and NOS-1) and decreased (NOS-2, Nfkb) gene expression (Mapk1 not activated) in rats that received BPC 157 therapy following reperfusion, as a way of showing how BPC 157 may act [145].

In the recovery of myotendinous junction defect (dissection of the quadriceps tendon from the quadriceps muscle in rats), BPC 157 increases *eNOS* mRNA levels and decreases *COX 2* mRNA levels during the whole 7–42-day period. Again, this effect seems to be related to disease conditions, as, in healthy rats, BPC 157 had no effect [80].

In the counteraction of the ketamine-induced models resembling negative-like symptoms of schizophrenia in rats, the evidenced effect on the given gene expression in the brain tissue of BPC 157 therapy is distinctive [171]. Applied immediately after ketamine, it exhibited effects on *NOS-1* (decreased expression), *NOS-2* (increased expression), *Plcg1* (decreased expression), *Prkcg* (increased, and then decreased expression), and *Ptgs2* (increased expression), and it had no effect on *NOS-3* and *Ptk2* [171]. These findings may indicate a timely, specific BPC 157 effect on ketamine-specific brain targets [171].

### 2.8. Summarizing BPC 157/Angiogenesis, Cornea Neovascularization, VEGF, and Tumor Growth

In summary, as was already emphasized, all of these beneficial effects can occur as general practical proof that with BPC 157 therapy, the cytoprotection concept and the concept of a general adaptive syndrome [41,42,43,44,45,46,47,48,49] might be reconciled with the established principles of homeostasis [3,4,5,6,7,8,9,10,11,12,13,14,16,17,18,19,20,21,22,23,24,27,28,29,30,31,32,34,37,39,40]. Likewise, as a discriminative point with BPC 157 therapy [3,4,5,6,7,8,9,10,11,12,13,14,16,17,18,19,20,21,22,23,24,27,28,29,30,31,32,34,37,39,40], angiogenesis, commonly sharing importance for cytoprotective agent activity [172,173,174], is tightly connected with healing depending on the organ involved [3,4,5,6,7,8,9,10,11,12,13,14,16,17,18,19,20,21,22,23,24,27,28,29,30,31,32,34,37,39,40]. As a particular healing capability has been demonstrated, BPC 157 effectively cures gastrointestinal ulcers, skin wounds, tendon and muscle lesions, and liver lesions (see, for review [16,21,118]), as well as corneal ulcers, and it maintains corneal transparency [76]. As pointed out [22], BPC 157 exerted its enhancement effects on the proliferation, migration, and tube formation of endothelial cells, for which phosphorylated levels of ERK1/2 were pivotal in this strong healing acceleration [92,93,94,100]. Consistent with Folkman (inhibited corneal neovascularization, inhibited tumor growth) [68,69,70], in the human melanoma cell line, it inhibits the VEGF effect [95], attributed to controlling the VEGF system, as well [92,93,94]. Furthermore, in mice with C26 colon adenocarcinoma, BPC 157 counteracted tumor cachexia and severe muscle wasting, corrected deranged muscle proliferation and myogenesis, counteracted weight loss, and markedly prolonged survival [22]. BPC 157 significantly counteracted an increase in proinflammatory and procachectic cytokines, such as interleukin 6 (IL-6) and TNF-alpha [22] (a similar effect also appeared in rats with bile duct ligation and cirrhosis treated with BPC 157 therapy [118]). Note that the pro-tumorigenic function of TNF and IL-6 is well-established [175,176]. The role of TNF and IL-6 as master regulators of tumor-associated inflammation and tumorigenesis makes them attractive targets for adjuvant treatment in cancer. Thus, it is likely that BPC 157 therapy inhibited promoting tumorigenesis [177], tumor growth [178], angiogenesis [178], and cancer cell invasion and metastasis [179] that would otherwise be induced [175,176]. With the beneficial effect of BPC 157 therapy, this can occur along with blocking VEGF signaling during the “angiogenic switch” and initial tumor growth [95] and the counteraction of severe adverse effects occurring with cytostatic drug applications [180,181,182], thereby improving their anti-tumor effectiveness. On the other hand, BPC 157 therapy is combined with the stimulation of anabolic pathways (FoxO3a, p-AKT, p-mTOR, and P-GSK-3β) [22].

Finally, as the presented evidence shows, controlling/modulating angiogenesis [3,4,5,6,7,8,9,10,11,12,13,14,16,17,18,19,20,21,22,23,24,27,28,29,30,31,32,34,37,39,40] should occur along with similar corresponding controlling/modulating of NO-system functioning [11,29,92,93,94,97,98,99] (see below, Section 3).

This controlling/modulating angiogenesis [3,4,5,6,7,8,9,10,11,12,13,14,16,17,18,19,20,21,22,23,24,27,28,29,30,31,32,34,37,39,40] and similar corresponding controlling/modulating NO-system functioning [11,29,92,93,94,97,98,99] occurred as particularities in BPC 157 therapy. An additional clue reviewed overcame the standard angiogenic growth factors for healing in the gastrointestinal tract and, particularly, for the healing of extra-gastrointestinal tissues (i.e., skin, tendon, ligament, muscle, bone) [16,21]. Namely, in general, in standard peptide therapy, there is a regular need for special and various delivery systems and various carrier additions [16,21]. Firstly, this obscures the inherent beneficial effect of any peptide that needs carrier addition or a special delivery system for its effects [16,21]. Then, there is general uncertainty about whether the peptide or the carrier or the peptide+carrier(s) complex might be essential for the obtained effect [16,21].

Together, these avoided pitfalls emphasize as a particular exception the stable gastric pentadecapeptide BPC 157 (native and stable in human gastric juice for more than 24 h), always given alone [3,4,5,6,7,8,9,10,11,12,13,14,16,17,18,19,20,21,22,23,24,27,28,29,30,31,32,34,37,39,40]. BPC 157’s beneficial effects, therefore, are regularly and unmistakably attributed and clearly established [3,4,5,6,7,8,9,10,11,12,13,14,16,17,18,19,20,21,22,23,24,27,28,29,30,31,32,34,37,39,40].

Therefore, as a particular follow-up, the particularities support each other, and the beneficial effects imply that particularly controlled angiogenesis could be a very likely positive outcome of BPC 157 therapy [3,4,5,6,7,8,9,10,11,12,13,14,16,17,18,19,20,21,22,23,24,27,28,29,30,31,32,34,37,39,40].

## 3. NO-System

All of these points may be supported by its special interaction with various molecular pathways [18,22,92,93,94,95,96,100,183,184,185,186]. This includes, in particular, the NO-system [11,29,92,93,94,97,98,99] as a whole. Evidence showed, as mentioned, wide therapy counteracting potential (NO-release, NO-synthase (NOS)-inhibition (L-NAME), NOS-over-activity (L-arginine), NO-system immobilization (L-NAME+L-arginine). Indicative therapy occurred for hypertension, hypotension, and thrombocytes’ function (without affecting the coagulation cascade) [11,29,97,98,99,187,188] and signaling pathways controlling vasomotor tone [92,93,94] (VEGFR2-Akt-eNOS and Src-Caveolin-1-eNOS). At the general level, this dual (modulatory) action (i.e., either hypertension or hypotension reversed toward normal blood pressure) applies to the NO-system’s effects as a whole, as well [11,29]. Such a role of BPC 157 therapy could be essential amid the dual role of NO, as both inhibition and an uncontrolled excess of NO could lead to significant damage [189,190]. Consequently, BPC 157’s ability to restore NO-system homeostasis may represent a central mechanism underlying its wide-ranging therapeutic potential [11,29].

Therefore, as reviewed [11,29], BPC 157 does not exhibit characteristics of a NO-system disruptor, NO-system dysregulation, or a negative chain of events [191]. As the work of Judah Folkman defined the induction of corneal neovascularization and angiogenesis that can lead to tumorigenesis [68], in the case of NO-system disturbances, as described by Moncada, the threatening chain of events is well-defined [191]. Agents should induce NO-overactivity and over-release, increased free radical formation, and severe disturbances (including neurodegenerative diseases) [191].

Firstly, BPC 157 regulates the NO-system [11,29]. Therefore, its stimulation of the NO-system is not simple, uncontrolled activation but rather a highly controlled and regulated effect. Indicatively, BPC 157 strongly opposed the NO-over-release induced by L-arginine [97,98]. It counteracted free radical formation regardless of the effect on NO, whether an increase or decrease [80,144,175,181,182,192,193,194,195,196,197]. The NO-level in tissue, whether increased or decreased, was regularly normalized through BPC 157 administration [11,29]. Finally, it strongly counteracted neurodegenerative diseases, including Parkinson’s disease and Alzheimer’s disease, in animal models [145,198,199,200]. In both theory and practice, given the beneficial effects obtained [11,29], the counteraction of free radical formation (along with any effect on NO-level or eNOS expression) and its beneficial effects in neurodegenerative diseases support each other’s effects. Thus, this evidence compellingly suggests the value of BPC 157 therapy. The essential tool (i.e., targeting NO’s cytotoxic and damaging actions but not interfering with its essential protective functions and even promoting them) can be implemented [3,4,5,6,7,8,9,10,11,12,13,14,16,17,18,19,20,21,22,23,24,27,28,29,30,31,32,34,37,39,40].

Secondly, there exists an extensive network of interrelated and beneficial healing effects associated with BPC 157 therapy, as reported in studies investigating BPC 157 and the NO-system [11,29]. More than 80 affected targets have been identified, including those resulting from NO-system blockade as well as those from NO-system overactivity (for a review, see [11,29]). This reveals distinctive “NO-system clusters” [3,11] that highlight distinct patterns of NO-related regulation and dysfunction [3,11] and distinctive NO-agents’ responses [3,11].

The following discussion will substantiate that BPC 157 neutralizes NO’s cytotoxic and damaging effects while supporting its beneficial and essential protective functions.

### 3.1. Multitude of BPC 157 Beneficial Effects Related to the NO-System as Proof of Concept

Accordingly, the previous review conceptualized BPC 157/NO-system relationships and BPC 157 therapy’s beneficial effects [29]. These included effects on (i) gastric mucosa and mucosal protection, following alcohol lesions, in cytoprotection course, NO-generation, and blood pressure regulation; (ii) alcohol acute/chronic intoxication and withdrawal; (iii) cardiovascular disturbances, chronic heart failure, pulmonary hypertension, and arrhythmias; (iv) disturbances after hypokalemia and hyperkalemia and potassium cell membrane dysfunction; and (v) complex healing failure, proved by the fistulas’ healing [29].

Furthermore, the greater the specific points identified where the two systems may interact, the closer the relationships [11]. The next studies revealed particular relationships with sphincter function [197], free-radical-induced injuries [197], bleeding [144,187,192], non-specific and specific NSAID-induced lesions [201], general anesthesia (thiopental) [202] and local anesthesia (lidocaine) [203] induced disturbances, and rat models that resemble schizophrenia-positive symptoms and negative symptoms [171,200]. Most importantly, with organ lesions or vessel occlusion, severe multiorgan and vessel failure, and occlusion/occlusion-like syndrome, there is a key therapy effect on the vessels’ presentation and recruitment of additional collateral pathways to bypass occlusion and reestablish reorganized circulation [129,130,131,132,133,134,135,136,137,138,139,140,141,142,145,168].

The way BPC 157 therapy interacts with the NO-system addresses various targets and the specific effects it has under different conditions. This provides a piece of collective evidence, various models, and a multitude of targets involving distinct NO-response patterns. These signify compelling, beneficial evidence about adequate recovery of NO-system functions through well-controlled BPC 157 therapy [11,29]. Furthermore, as mentioned, BPC 157’s overwhelming action in general resolved the simultaneous application of NO-agents given alone, including L-NAME (NOS-blockade) and L-arginine (NOS-substrate), or together, including L-NAME+L-arginine, NO-inhibition, NO-overactivity, and NO-immobilization. This effect’s complexity (distinct NO-response patterns) includes their distinctive relationships, requiring the investigation of NO-system function as a whole, with all of its particularities, to realize the BPC 157/NO-system relationship [11,29]. As mentioned, a collection of more than 80 distinctive targets revealed many distinctive NO-system presentations at the investigated targets, giving many distinctive typical responses of NO-agents [11,29] and distinctive NO-system clusters [3,11,29] (Table 3). These were L-NAME responsive/L-arginine responsive, L-NAME responsive/L-arginine non-responsive, L-NAME non-responsive/L-arginine responsive, opposite or parallel, NO-specific (L-NAME and L-arginine counteract each other’s response), or NO-non-specific (L-NAME and L-arginine do not counteract each other’s response) (Table 3). Thus, the beneficial effect of BPC 157 therapy occurs in distinctive NO-system circumstances (given that each NO-agent application reflects particular endogenous circumstances), occurring regularly with similar beneficial effects [11,29].

Therefore, a multitude of BPC 157 beneficial effects related to the NO-system presented proof of concept [11,29] that BPC 157 consistently counteracted the induced general dysregulation of the NO-system, uncontrolled NO increase, and free radical formation and finally counteracted the course leading to neurodegenerative diseases. Likewise, BPC 157 consistently counteracted the induced general dysregulation of the NO-system, such as NO-system blockade.

To specify the BPC 157–NO-system relationship and effects, we introduced as a highlight matching between the effects (i.e., L-NAME non-responsive/L-arginine responsive (L-NAME NR, L-arginine R, *opposite, specific*)). The matching between the effects can indicate matching between the involved targets and therefore “mapping” of the NO-system in the body by NO-agents’ common or distinctive effects and relationships [11,29].

Using the “L-NAME non-responsive/L-arginine responsive” paradigm, an indicative example is rat models that resemble schizophrenia-positive symptoms and negative symptoms [171,200]. BPC 157 therapy counteracted cognitive dysfunction in a novel object recognition test [171], particularly resembling a “negative-like” symptom [204,205,206,207]. Likewise, BPC 157 therapy counteracted similar “positive-like” symptoms [200] (acute apomorphine, chronic methamphetamine, and acute MK-801 induced effects and acute haloperidol induced catalepsy) [208,209,210,211,212,213,214,215]. For the possible but so far not recognized BPC 157/NO-system connection, we indicated [171] that under the same “L-NAME non-responsive/L-arginine responsive” paradigm, this counteraction of resembling “negative-like” symptoms (cognitive dysfunction) [204,205,206,207] completely corresponded to the counteraction of the similar “positive-like” symptoms [200] (acute apomorphine, chronic methamphetamine, and acute MK-801 induced effects and acute haloperidol induced catalepsy) [208,209,210,211,212,213,214,215]. Therefore, the same NO-therapy effect might indicate a particular match between the similar “negative-like” symptoms and the similar “positive-like” symptoms and shared NO-pathology [205,206,207,208,209,210,211,212,213,214,215]. Generally, in “L-NAME non-responsive, L-arginine responsive” NO-response in both circumstances, BPC 157’s beneficial therapeutic effect (antagonization) goes over L-arginine (antagonization). BPC 157 therapy counteracted all of the symptoms that resembled “positive-like” and “negative-like” symptoms [171,200].

Likewise, BPC 157 therapy counteracted the negative consequences of hyperkalemia, hypokalemia, hypermagnesemia, and hyperlithemia, which appear as distinctive NO-system clusters (hyperkalemia, hypokalemia cluster vs. hypermagnesemia, hyperlithemia cluster) [3]. Note that the used triple NO-agents’ application and BPC 157’s application as a simple but useful NO-key [11,29] shared the same dose relationship (L-NAME (5 mg/kg), L-arginine (100 mg/kg), BPC 157 (10 µg/kg)) in all BPC 157/NO-studies (for a review, see [11,29]). This might be seen as a network of evidence for the physiologic significance of the revealed BPC 157/NO-system interplay (i.e., BPC 157 was found in in situ hybridization and immunostaining studies in humans to be largely distributed in tissues [20] and may have additional physiologic regulatory roles [3,4,5,6,7,8,9,10,11,12,13,14,16,17,18,19,20,21,22,23,24,27,28,29,30,31,32,34,37,39,40]).

Thus, BPC 157 has beneficial and overwhelming effects over NO-agents. They occur pleiotropically in distinct ways and, depending on the involved target and NO-agents’ relationship presentation [11,29], BPC 157 therapy consistently acts to reestablish normal circumstances (normotension (BPC 157) vs. hypertension (L-NAME) vs. hypotension (L-arginine) [152]; normal behavior (BPC 157) vs. catalepsy (haloperidol) vs. stereotypes (amphetamine) [200]). There were mostly L-NAME’s and L-arginine’s opposite effects and, less frequently, L-NAME’s and L-arginine’s parallel effects [11,29] (Table 2). In addition to amphetamine’s effect [197], this parallelism occurs with quite distinctive models (miosis, atropine–mydriasis [111], huge magnesium overdose [216], ischemic/reperfusion colitis [194], duodenal congestion lesions [193], cecum perforation [192], and L-NAME and/or L-arginine interaction with other systems (i.e., acetylcholine) [111]). BPC 157 therapy’s application counteracted all of these effects [11,29]. Finally, BPC 157 therapy can also promptly counteract severe multiorgan and vascular failure that could be induced by applying NO-donors, like isosorbide mononitrate, in rats (report in preparation).

Therefore, ischemic/reperfusion colitis (medication (BPC 157, L-NAME, L-arginine (alone/combined), saline, as a bath at the blood-deprived colon segment by two ligations of the left colic artery and vein) appears to be an additional illustrative complex example of distinctive NO-response patterns depending on the target [194]. While BPC 157 provided a consistent beneficial effect (10 μg/kg bath (1 mL/rat) increased vessel presentation with inside/outside arcade interconnections’ quick reappearing, mucosal folds’ preservation, and the pale areas becoming small and markedly reduced), NO-agents have a more complex effect. L-NAME initially caused all vessels to disappear more rapidly and L-arginine increased the number of vessels (L-NAME R, L-arginine R, *opposite*), but both induced larger pale areas (L-NAME R, L-arginine R, *parallel*), all as NO-specific (NO-related) effects, as L-NAME and L-arginine regularly attenuated or antagonized each other’s responses (L-NAME R, L-arginine R, *opposite*, *specific*; L-NAME R, L-arginine R, *parallel*, *specific*).

In summary, consistent studies have fully elaborated the negative evidence (i.e., BPC 157 counteracted worsening effects induced by L-NAME) [11,29]. Likewise, the same studies have also fully elaborated the positive evidence (i.e., BPC 157 counteracted worsening effects induced by L-arginine) [11,29]. Finally, in the same way, there has been simultaneous elaboration of the third form, which is neutral evidence (i.e., BPC 157 counteracted the remaining serious pathology in the animals treated with L-NAME + L-arginine, L-NAME (NO-blockade) vs. L-arginine (NO-over-stimulation) (opposing each other’s response) = control) [11,29]. Together, whatever the mechanism background, this indicates that the BPC 157 system functions along with the NO-system [11,29]. As experimental evidence has demonstrated, it will reactivate the NO-system once inactivated by the combined action of L-NAME and L-arginine. Counteraction of the remaining serious pathology in the animals treated with L-NAME + L-arginine could mean restoration of NO-system function again, coinciding with BPC 157’s additional application (L-NAME + L-arginine+BPC 157). Thus, BPC 157 therapy was effective regardless of whether the NO-system was inactivated (L-NAME + L-arginine), overstimulated (L-arginine), or blocked (L-NAME) [11,29]. Thus, in this particular case of ischemic/reperfusion colitis, this may be a consolidation of the stimulatory and inhibitory effects of the NO-system to produce more effective healing (i.e., by promoting the interconnection of arcade vessels to bypass major obstructions) [194]. Furthermore, given that BPC 157 induces the same effect as therapy applied during reperfusion, or even later, in rats that had severe bowel obstruction, BPC 157 therapy could be a fundamental treatment involving the NO-system. It quickly restored blood supply to the ischemically injured area and rapidly activated collaterals. This occurred along with the recovery of the NO-level to normal values through BPC 157 therapy (regularly, the NO-level decreased during ischemia and increased during reperfusion) and counteraction to normal values of the increased MDA values (increased in ischemia and even more in reperfusion) [194] (see Section 3.2).

### 3.2. NO-Level in Tissue, Increased or Decreased, Was Regularly Normalized Through BPC 157 Administration, Along with Increased MDA-Level Decrease (and/or Normalization) Through BPC 157 Administration

As emphasized before [11,29], the BPC 157/eNOS relationship is highly related to ongoing healing (and therefore increased, decreased, or not affected) (see Section 2.7), as and the effect of the NO-level in tissue, whether increased or decreased, is regularly normalized by BPC 157 administration, along with decreasing (and/or normalizing) the increased MDA-level.

Most likely, given consistent beneficial effects, BPC 157 therapy could specifically counteract the cytotoxic and damaging actions of NO, being organ-specific. As mentioned, through increased MDA values and decreased NO-values, BPC 157 reversed values to normal, healthy values (perforation, severe ischemic/reperfusion colitis) [144,192,193,194]. Counteraction of the increased NO and MDA levels occurred with vessel occlusion, cirrhosis, cytostatic application, and haloperidol application [118,181,182,194,195,196,197].

Similarly, the effect of BPC 157 therapy in rats with myotendinous junction defect was recovery, along with counteraction of the increased MDA values and the increased NO values in the myotendinous junction [80]. Finally, in rats with infrarenal occlusion of the inferior caval vein, the beneficial effect of BPC 157 therapy includes counteraction of the increased MDA values and counteraction of an increase in the NO-level in plasma and the inferior caval vein [129].

In support, there is a piece of additional ample evidence that BPC 157 acts as a free radical scavenger in many distinctive models and organs and thereby exerts its pleiotropic effect (i.e., [18,22,80,90]), which is relevant in prompt reversal in vascular occlusion/occlusion-like failure studies [129,130,131,132,133,134,135,136,137,138,139,140,141,142,168]. In addition, the increased MDA values’ counteraction occurred in all organs simultaneously, as specifically shown in the therapeutic effect investigated in rats that had intra-abdominal hypertension (grade III and grade IV), decompression, and reperfusion [133,134]. The consistent decrease in increased MDA values in the blood, brain, heart, liver, kidney, and gastrointestinal tract is the next piece of evidence that BPC 157 therapy exerts its pleiotropic beneficial effects in all rats that have intra-abdominal hypertension (grade III and grade IV), decompression, and reperfusion in a particular way [133,134]. This may occur as a result of damage to the vascular wall—note that the highest MDA values were observed in the blood—and in other tissue cells, particularly in the presence of reactive oxygen intermediates and impaired endothelial function [133,134]. Such a therapeutic effect, which simultaneously targets all of these mechanisms, could be a long-overdue breakthrough in the treatment of acute abdominal compartment syndrome [217]. This is especially significant given the challenges and limitations [217] that have been difficult to overcome with previous approaches. An additional point is radiation-induced liver disease, the major complication for cancer patients after radiation therapy [183]. The therapeutic effects of pentadecapeptide BPC 157 occurred in reducing radiation-induced liver disease through Kruppel-like factor 4 upregulation both in vivo and in vitro [183].

It is noteworthy that such beneficial effects (counteraction of increased MDA values along with the distinctive effect on NO and eNOS) and a general lack of toxicity [3,4,5,6,7,8,9,10,11,12,13,14,16,17,18,19,20,21,22,23,24,27,28,29,30,31,32,34,37,39,40] would also be instructive for all metabolites, and therefore metabolites would also participate in the noted beneficial effects (see Section 1).

### 3.3. BPC 157 and Parkinson’s Disease and Alzheimer’s Disease

Commonly, NO has a well-known dual potential, i.e., neuroprotective potential in some contexts and neurodegeneration if produced excessively or in the wrong context. Therefore, for Parkinson’s disease and Alzheimer’s disease, it plays a pivotal role in both protection and exacerbation [218,219,220,221,222,223,224].

In practice, such a controlling, modulatory role was ascribed to BPC 157 therapy application as a particular action [11,29] (see Section 3.1 and Section 3.2). As emphasized, this was based on the compelling evidence collected from various models, a multitude of targets, and BPC 157’s overwhelming action versus the simultaneous application of NO-agents, whether given alone, including L-NAME (NOS-blockade) and L-arginine (NOS-substrate, NO-over-stimulation), or together, including L-NAME+L-arginine, NO-inhibition, NO-overactivity, and NO-immobilization. Therefore, investigation of NO-system function as a whole could help realize the BPC 157/NO-system relationship [11,29].

**Figure 3:**
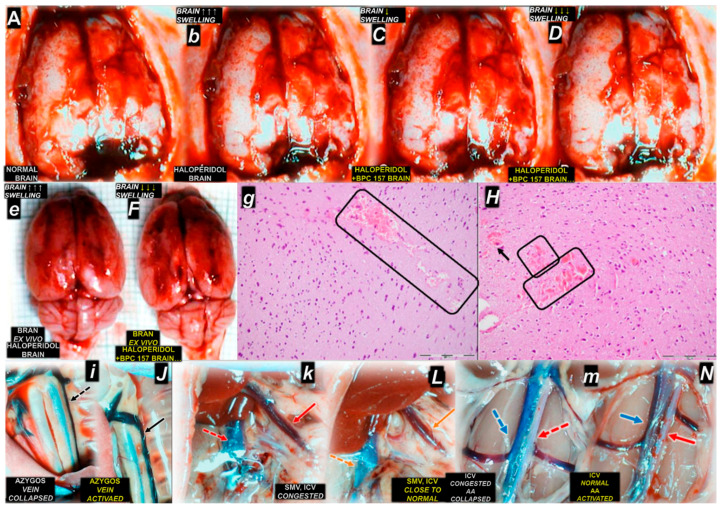
Illustrative course of 15 min following haloperidol in rats, occlusion/occlusion-like syndrome, and antecedent behavioral changes and the counteracting effect of BPC 157 therapy (10 µg/kg, 10 ng/kg ip or ig) given at 5 min after haloperidol (5 mg/kg ip) (**A**–**N**) (normal capital letter for healthy, *small italic letters* for control haloperidol, *capital italic letters* for haloperidol counteracted by BPC 157 therapy) [142]. Calvarial window brain presentation before the challenge (**A**), after haloperidol challenge (**b**) application, and after the therapy’s application (**C**,**D**), which counteracted brain swelling after BPC 157 application (*italic capital letters*): immediately upon BPC 157 administration (***C***), and further decreased brain swelling in the BPC 157 treated rat immediately before sacrifice (***D***). Upon sacrifice at 15 in following haloperidol, brain swelling in haloperidol rats (**e**) was counteracted by BPC 157 therapy (**F**). In microscopy studies, HE, magnification 200×, pronounced edema and congestion were visible, affecting the cerebrum, and there was more prominent intracerebral cortical hemorrhage involving larger areas of cerebral brain tissue affecting the neocortex (rectangle area) (**g**). BPC 157 (***H***). Only mild edema and congestion were found, with small, focal, and superficial areas of neocortical hemorrhage (rectangular areas and black arrow). Illustrative presentation of the recovery of vascular failure (arrows) presenting a collapsed azygos vein in haloperidol rats (**i**), recovered to an activated azygos vein (and, therefore, azygos vein direct blood flow delivery) in BPC 157 rats (**J**). Furthermore, the superior mesenteric vein (SMV) and inferior caval vein (ICV), which were congested in haloperidol rats (**k**), recovered to normal vein presentation in BPC 157 rats (**L**). Likewise, the inferior caval vein (ICV) was congested and the abdominal aorta (AA) collapsed in haloperidol rats (**m**); the inferior caval vein (ICV) recovered to normal presentation, and the abdominal aorta (AA) appeared activated in BPC 157 rats (**N**). An illustrative outcome was the evidence that BPC 157 therapy counteracted occlusion/occlusion-like syndrome as a whole for each organ involved, including brain, heart, lung, liver, kidney, and gastrointestinal tract lesions and hemorrhage, and did not produce any adverse effects on account of its beneficial effects. Moreover, intracranial (superior sagittal sinus), portal, and caval hypertension, aortal hypotension, and thrombosis were eliminated/attenuated, and thus advanced Virchow triad circumstances were fully reversed. Similar beneficial results were obtained in the counteraction of occlusion/occlusion-like syndrome induced by other neuroleptics (i.e., fluphenazine, clozapine, risperidone, olanzapine, quetiapine, and aripiprazole, but also domperidone and amphetamine [142].

As pointed out [224], there are many relevant animal models for the study of Parkinson’s disease. These are neurotoxin induction-based models (1-methyl-4-phenyl-1,2,3,6-tetrahydropyridine (MPTP), 6-hydroxydopamine (6-OHDA) and agricultural pesticides (rotenone, paraquat)), pharmacological models (reserpine or haloperidol treated rats), and genetic models (α-synuclein, leucine-rich repeat kinase 2 (LRRK2), DJ-1, phosphatase and tensin homolog (PTEN)-induced kinase 1 (PINK-1) and Parkin). As a highly relevant translation, MPTP was first known to produce severe parkinsonism in humans (i.e., since 1982) [225]. Thus, considering BPC 157/Parkinson’s disease/Alzheimer’s disease, BPC 157 therapy was effective in Parkinson’s disease models, and it counteracted parkinsonogenic neurotoxin MPTP-induced tremor, rigor, akinesia, and gastric lesions and counteracted mortality in mice, reserpine-induced akinesia, catalepsy, hypothermia, and catalepsy induced by neuroleptics or NOS-blocker L-NAME application [198,199,200]. For counteraction of haloperidol and other neuroleptic-induced disturbances since their early course, see counteraction of occlusion/occlusion-like syndrome, severe multiorgan and vascular failure, as a whole, by particular vascular recovery and activation of rescuing collateral pathways (i.e., activation of azygos vein direct blood flow delivery) [4,10]. As an insight, there is a study of innate vascular failure caused by application of neuroleptics, amphetamine, and domperidone that rapidly induced severe occlusion/occlusion-like syndromes in rats and stable gastric pentadecapeptide BPC 157 as therapy [142] (Figure 3).

Consistently, BPC 157 induces the release of serotonin in specific brain nigrostriatal regions and influences serotonergic and dopaminergic systems [226]. Indicatively, with an additional dopamine-regulating effect, BPC 157 counteracted the models of the positive-like and negative-like similar symptoms in schizophrenia rat models [171,200]. Also, BPC 157 counteracted various motor disturbances and muscle disabilities, along with the counteraction/amelioration of the prime cause (for a review, see [12,13]). These muscle disabilities were induced by different peripheral causes (e.g., direct muscle, tendon, ligament, and nerve injury [80,81,82,83,84,85,86,87,88,89,90,91], succinylcholine [227], vascular occlusion [228], over-dose of potassium, magnesium or lithium [138,216,229], and tumor cachexia [22]). Likewise, in addition to the mentioned Parkinson’s disease models [198,199,200], these were induced by various central causes (e.g., spinal cord compression [170,230], stroke [145], traumatic brain injury [231], neurotoxin cuprizone that mimics multiple sclerosis in rats [232], neuroleptics [199,200], amphetamine [200,233], alcohol acute and chronic intoxication [234], serotonin syndrome [235], and NO-system blockade [200]). Interestingly, various sphincter failures [10,64,110,111,113,180] were recovered, and glaucoma-mydriasis [110,113], atropine-mydriasis, L-arginine-induced prolonged miosis, and L-NAME-induced prolonged miosis [111] were counteracted. Thus, we exemplified with BPC 157 therapy many targets—muscular, vascular, nerve, peripheral, and central—that share functions with the multimodal muscle axis [12,13].

Notably, ischemic brain episodes, like Alzheimer’s disease, mostly present alterations in the hippocampus [236]. Following bilateral clamping of the common carotid arteries during 20 min of ligation, as assessed at 24 h and 72 h of reperfusion, BPC 157 therapy counteracted both early and delayed neural hippocampal damage, achieving full functional recovery (Morris water maze test, inclined beam-walking test, lateral push test) [145]. As reperfusion therapy [145], there was a combined effect on particularly vulnerable hippocampal neurons [236], counteracting both early and delayed neural damage [145]. Achieving full functional recovery (based on the Morris water maze test, inclined beam-walking test, and lateral push test) means that functions were generally maintained after BPC 157 treatment. Without BPC 157 therapy, there were severely impaired locomotion capabilities, including a lack of forelimb and hindlimb motor coordination and resistance to lateral pushes from either side of the shoulder [145]. Likewise, fully maintained spatial learning and memory of the rats in the Morris water maze test was ascribed to hippocampal synaptic plasticity and NMDA receptor function [237] involving the entorhinal and perirhinal cortices, the prefrontal cortex, the cingulate cortex, the neostriatum, and perhaps even the cerebellum in a more limited way [237].

There is also the counteraction of cognitive dysfunction exemplified through the novel object recognition test as a particular “negative-like” symptom [204,205,206,207] in the schizophrenia model (acute ketamine treatment (3 mg/kg i.p.)) [171]. Likewise, with BPC 157 therapy, there is a consistent counteraction of hippocampal lesions that occurred in rats with an occluded major vessel, peripherally and centrally, which underwent major noxious procedures or damaging agent application as a consequence of severe multiorgan and vessel failure and occlusion/occlusion-like syndrome [129,130,131,132,133,134,135,136,137,138,139,140,141,142,168].

Finally, BPC 157 therapy can specifically and beneficially affect all brain structures (and, consequently, neurotransmitter disabilities) [6,9], with the areas most affected by lesions most presented. These were in the cerebellum (ibuprofen [238], paracetamol [239]), the cerebral cortex (diclofenac [240,241], celecoxib [201]), the frontoparietal cortex (concussive brain trauma) [231], the hippocampus and the cerebral cortex (insulin) [242], and the parietal neocortex and the hippocampus (cuprizone [232]). Note that cuprizone application [232] was an extremely high regimen and much higher than those commonly applied to mimic multiple sclerosis in rats [243,244]).

Therefore, it is evident that the explanation of the NO-system/BPC 157 relationship should be further extended [11,29]. Indicatively, there is activation of the VEGFR2-Akt-eNOS signaling pathway without the need for other known ligands or shear stress and evidenced control of vasomotor tone by the activation of the Src-Caveolin-1-eNOS pathway; there is also the relaxation of large vessels (ex vivo), mainly by acting on vascular endothelial cells but also on vascular smooth muscle cells [93,94]). Furthermore, in the vessel wall, there is a rapid change in the lipid contents and protein secondary structure conformation produced instantly via BPC 157 therapy [143] (Fourier transform infrared spectroscopy), supporting vessel function even in the worst circumstances. But, the strong beneficial effects have been compellingly demonstrated in various animal models, and these effects can speak for themselves [11,29]. As such [3,4,5,6,7,8,9,10,11,12,13,14,16,17,18,19,20,21,22,23,24,27,28,29,30,31,32,34,37,39,40], they would fully eliminate concerns extended to Parkinson’s disease and Alzheimer’s disease by Józwiak and collaborators’ review [1].

Likewise, given Parkinson’s or Alzheimer’s disease as NO-system-related disturbances, the NO-system/BPC 157 relationship could not contribute to their development. In contrast, Parkinson’s disease’s or Alzheimer’s disease’s relationship to the NO-system can compellingly explain the noted beneficial effects of BPC 157 in corresponding models, as described before.

In summary, the pleiotropic effects of BPC 157 therapy and the overall significance of the NO-system can have a particular modulatory/controlling capacity, consequently excluding the uncontrolled NO-system outcome (and therefore, i.e., Parkinson’s disease) [11,29]. As an analogy and a proof of concept, this fully substantiated the particularities of therapy outcomes in schizophrenia-like models [171,200]. As noted, BPC 157 therapy acting via NO-system distinctive presentations previously specified (i.e., L-NAME-responsible/L-arginine responsible, L-NAME-non-responsible/L-arginine-responsible) counteracted opposite disturbances (catalepsy (haloperidol) vs. stereotypes, increased locomotor activity (amphetamine, apomorphine)) [200]. Likewise, acting through the NO-system, BPC 157 counteracted various opposite disturbances, i.e., prolonged miosis and prolonged mydriasis [111], consequences of hyperkalemia and hypokalemia [3], hypertension and hypotension [152], and bleeding and thrombosis [129,130,131,132,133,134,135,136,137,138,139,140,141,142,168].

Finally, as additional support for such a particular beneficial effect of BPC 157 therapy, its cytoprotective effect is associated with the mitigation of leaky gut syndrome [18]. There was increased tight junction protein expression (e.g., zonula occludens-1 (ZO-1)) and transepithelial resistance, which significantly mitigates leaky gut syndrome and stabilizes cellular junctions [18]. Such stabilization of cellular junctions [18] further supports specific therapeutic effects. This is the inhibition of inflammatory mediators (e.g., inducible NO synthase (iNOS), IL-6, interferon-gamma (IFN-γ), and TNF-α) [18]. Simultaneously, there is an enhancement of the expression of heat shock proteins (e.g., HSP70 and HSP90) and antioxidant proteins (e.g., heme oxygenase (HO-1), NAD(P)H dehydrogenase (NQO-1), glutathione reductase, glutathione peroxidase 2, and glutathione S-transferase pi (GST-pi)) [18].

In conclusion, the resolution of leaky gut syndrome (a core mechanism underlying numerous systemic diseases) through BPC 157 therapy is a decisive argument that provides compelling evidence for the validity of its beneficial effects and supports the broader concept of cytoprotection [18] now described. Leaky gut syndrome is now recognized for its role in numerous systemic diseases, including digestive diseases [245], cardiovascular diseases [246], Alzheimer’s disease, depression, Parkinson’s disease [247,248], celiac disease [249], liver diseases [250] and renal diseases [251], metabolic syndrome [251], type 1 diabetes mellitus [252,253], and cancer [254,255].

## 4. Final Considerations Regarding General Concerns

Notably, the specific points discussed before might still indicate general concern [1]. Such concern encompasses the complexity and multifaceted nature of BPC 157’s biological activity and its interaction with multiple systems in the body. Therefore, in contrast with cytoprotection conceptualization [41,42,43,44,45,46,47,48,49], there might be a claim of an unescapable risk of unanticipated adverse effects of BPC 157 therapy due to its pleiotropic effects [1]. Specifically, the compound’s extensive biological activity should inherently entail an irreducible risk of unanticipated off-target or systemic adverse effects [1].

Thus, as a general understanding, in the context of experimental therapeutics, increasing model complexity—characterized by the engagement of multiple molecular targets and biological pathways—has to be associated with a proportional increase in the risk of adverse effects [1]. Given the pleiotropic beneficial effects of BPC 157, the complexity of the biological systems involved, and the potential risks associated with BPC 157, especially in terms of its interactions with multiple targets across different organs and tissues, the potential for unanticipated adverse effects should be greater. However, this is the case if the negative principle is not resolved.

Illustrative examples of the resolved principle in both ischemia and reperfusion conditions are the studies of BPC 157 therapy’s effect on severe multiorgan (brain, heart, lung, liver, kidney, and gastrointestinal tract lesions) and vascular failure and occlusion/occlusion-like syndrome [129,130,131,132,133,134,135,136,137,138,139,140,141,142,168]. These were carried out in rats with occluded major vessel(s), peripherally [129,130,131,132] or centrally [133], which underwent similar noxious procedures [134,135,136,137] and various damaging agent applications [138,139,140,141,142,168]. These conditions may be lethal, including severe arrhythmias, thrombosis, hemorrhage, and disturbed blood pressure (e.g., intracranial hypertension, portal hypertension, caval hypertension, and aortic hypotension), commonly in advanced Virchow triad circumstances [129,130,131,132,133,134,135,136,137,138,139,140,141,142,168]. Stable gastric pentadecapeptide BPC 157 [129,130,131,132,133,134,135,136,137,138,139,140,141,142,168] has proven its effectiveness in resolving these harmful events, counteracting brain, heart, lung, liver, kidney, and gastrointestinal tract lesions, arrhythmias, hemorrhage (brain, lung), and thrombosis, peripherally and centrally, intracranial hypertension, portal hypertension, caval hypertension, and aortic hypotension, which are markedly attenuated or even eliminated, with advanced Virchow triad circumstances fully reversed. The therapy as a shared effect, noted in the severe ischemia condition, as well as in advanced reperfusion, consistently and rapidly counteracts occlusion/occlusion-like syndrome as a whole, restoring function by effectively bypassing loops of minor vessels (e.g., direct blood flow [129,130,131,132,133,134,135,136,137,138,139,140,141,142,168] via the azygos vein) to counteract the failure of major vessels [129,130,131,132,133,134,135,136,137,138,139,140,141,142,168].

Therefore, as an expected extension of the theoretical framework concerning adverse effects [1], the extensive model complexities and the involvement of a multitude of biological targets [129,130,131,132,133,134,135,136,137,138,139,140,141,142,168] should, axiomatically, be associated with the emergence of adverse outcomes. Contrarily, there was a complete absence of such adverse outcomes. Now, this theoretical assumption as a paradigm instead supports that BPC 157 therapy exerts a highly coordinated therapeutic action. This can proceed as the advantageous application of the principle of vascular cytoprotection [129,130,131,132,133,134,135,136,137,138,139,140,141,142,168]. Indeed, the employed model’s complexity (occlusion/occlusion-like syndrome) [129,130,131,132,133,134,135,136,137,138,139,140,141,142,168] is very high. The consistent demonstration of therapeutic efficacy goes across the occluded vessel, peripheral and/or central, and similarly noxious procedures, as well as various damaging agents’ applications and a multitude of targets involved. In practice, this beneficial effect provides strong indication that BPC 157 therapy’s effects could resolve even the essential issue (i.e., Virchow triad circumstances, hemorrhage, and thrombosis) and that they are safe concerning each organ involved, including the brain, heart, lung, liver, kidney, and gastrointestinal tract, and do not produce any adverse effect on account of its beneficial effects [129,130,131,132,133,134,135,136,137,138,139,140,141,142,168]. Furthermore, the observed absence of adverse effects across multiple organ systems—including the brain, heart, lungs, liver, kidneys, and gastrointestinal tract—strengthens the evidence for its safety profile and supports its systemic cytoprotective potential [129,130,131,132,133,134,135,136,137,138,139,140,141,142,168].

This conclusion supports an additional, most recent study demonstrating that BPC 157 exerts a significant protective effect against distant organ damage in the liver, kidneys, and lungs following lower extremity ischemia-reperfusion injury in rats [256].

In conclusion, to counteract the tentative theoretical concerns about BPC 157’s complexity and multiple biological interactions, the extensive evidence provided by existing studies strongly supports the peptide’s safety and efficacy in resolving severe and multifactorial health issues without causing adverse effects [3,4,5,6,7,8,9,10,11,12,13,14,16,17,18,19,20,21,22,23,24,27,28,29,30,31,32,34,37,39,40].

## 5. Human Data

In comparison with animal data, current human studies are still scarce.

A study frequently mentioned in our studies [3,4,5,6,7,8,9,10,11,12,13,14,16,17,18,19,20,21,22,23,24,27,28,29,30,31,32,34,37,39,40] was a multicenter, randomized, double-blind, placebo-controlled study [257], which was performed to assess the efficacy, safety, and pharmacokinetics of BPC 157 in patients with mild to moderate ulcerative colitis. A total of 53 patients were randomized in a 1:1 ratio to receive BPC 157 enema, 80 mg once daily for 2 weeks, or placebo. The primary efficacy end point was a change in Disease Activity Index (DAI) over the treatment period. The DAI was defined as a composite score of clinical, laboratory, endoscopic, and pathohistological findings. Unlike the placebo, BPC 157 induced a statistically significant decrease in the DAI at the end of a 2-week treatment period and reduced the mean stool frequency, improved stool consistency, and had beneficial effects on histopathological findings. BPC 157 was very well-tolerated and safe. There was no difference in the frequency or type of adverse events in comparison with the placebo. BPC 157 was not detected in any of the plasma samples [257].

Many animal studies [3,4,5,6,7,8,9,10,11,12,13,14,16,17,18,19,20,21,22,23,24,27,28,29,30,31,32,34,37,39,40], following the reported improved mild to moderate ulcerative colitis in patients, revealed considerable effectiveness in various models (ulcerative colitis, ulcerative colitis complicated with anastomosis, external and internal fistulas, short bowel syndrome). Likewise, remarkable safety (i.e., LD50 higher than 2000 mg/kg through oral or intravenous route in mice) confirmed no adverse effect in toxicology studies (none of the animals died). In addition, included were, i.e., 4-week intravenous toxicity in rats followed by 4 weeks of recovery, 4-week intravenous toxicity in dogs, sub-acute (14 days) intracolonic toxicity study in rats and Beagle dogs, a repeated dose (28 days) dermal toxicity study in Wistar rats, chromosome aberrations in human lymphocytes cultured “in vitro”, an Ames test, a micronucleus test, female fertility and early embryonal development after intravenous administration to rats, embryo–fetal development after intravenous administration to rats (including toxicokinetics in pregnant animals and placental transfer), embryo–fetal development after intravenous administration in New Zealand White rabbits, cardiovascular and respiratory systems in anesthetized dog, a sensitization study in guinea pigs (Maximization test), and an acute eye irritation/corrosion study in albino rabbits.

An absorption, distribution, and excretion study of BPC 157 was performed in rats with the test article ^3^H-PL-10.1.AK-15 (^3^H-PL-10.1.AK-15. Pharmacokinetics in the rat after single oral administration. Istituto di Ricerche Biomediche “A. Marxer”, RBM: 3 May 1996) (Table 4).

At 168 h after administration, 12.0 ± 1.1% of the dose was recovered in feces. Given the observed rates of excretion in urine and feces, about 20–25% is expected to be present in the organism at 168 h after administration, and about 30–35% of the dose has probably been eliminated given ^3^H20 in expired air after metabolization of the test compound and isotope exchange with body water.

These findings are generally similar to the presentation of Xu and colleagues [258] and He and colleagues [259].

In phase I, a explorative, single-blind, placebo-controlled study [260] was conducted to assess the safety, tolerability, and preliminary pharmacokinetics of BPC 157 in thirty-two healthy male volunteers (A placebo-controlled study to investigate the safety, tolerability and preliminary pharmacokinetics of PL 14736 in healthy male subjects. FOCUS Clinical Drug Development GmbH; FOCUS Report 20PV0673; 2001). BPC 157 was administered in a form of rectal solution given through an enema container at four different dose levels: 0.25 mg/kg, 0.5 mg/kg, 1 mg/kg, and 2 mg/kg. Single and repeated intracolonic doses of BPC 157 to healthy male volunteers were very well-tolerated. No difference in placebo dosing was observed for any of the safety parameters measured. Because most BPC 157 plasma concentration profiles were below the assay LLQ at all time points, it seems that very little BPC 157 is absorbed into systemic circulation following rectal administration [260].

These findings are generally similar to the presentation of the Phase 1 Clinical Trial (42 volunteers) (NCT02637284), which established that the oral self-administration of BPC 157 in doses of 1, 3, 6, or 9 mg per day for as long as two weeks is safe and well-tolerated. No quantifiable amounts of BPC 157 in the volunteers’ plasma and urine samples were detected for the different dose regimes of Phase 1A and Phase 1B.

The study performed at a private clinic in Florida, which was IRB-approved, was conducted to assess whether intravenous BPC-157 is safe in humans [261]. Baseline blood work and vital signs were obtained from two participants before and after each infusion. On day 1, 10 mg of BPC-157 in 250 cc of normal saline was infused over one hour. On day 2, fasting blood work was repeated, vital signs were recorded, and 20 mg of BPC-157 in 250 cc of normal saline was infused over one hour. On day 3, fasting blood work and vital signs were repeated. Patients were questioned about any side effects at each appointment. The infusions of BPC-157 resulted in no measurable effects on the tested biomarkers of the heart, liver, kidneys, thyroid, or blood glucose levels. The BPC-157 peptide infusion was tolerated, with no side effects reported.

Many animal studies have revealed the beneficial effect of BPC 157 therapy on muscle, tendon, ligament, and bone healing [80,81,82,83,84,85,86,87,88,89,90,91]. In particular, there is evidence that pentadecapeptide BPC 157 given intraarticularly counteracts knee osteoarthritis in rats [262]. Along with these findings, a small study suggests that intra-articular injection of BPC-157 helps with multiple types of knee pain in patients, as well [263].

Intra-articular injection of BPC 157 for multiple types of knee pain was the focus of a retrospective study (16 patients, a 1-year chart review from 2019 to 2020), which was performed at the Institute for Hormonal Balance in Orlando, Florida, USA, to see whether intra-articular injection of the peptide BPC 157, alone or combined with TB4, helped relieve knee pain. Twelve had received only BPC 157 as an intra-articular injection (BPC 157 2 mg–4 mg). Eleven of the twelve patients (91.6%) had a significant improvement in knee pain, and, in most of them, the improvement lasted between 6 months and 1 year and they enjoyed improved sleep as well as mobility when knee pain decreased. There was no physical therapy after the peptide injection [263].

Assessment of the safety and efficacy of BPC-157 as a treatment for interstitial cystitis was the focus of the study carried out on 12 women who had not responded to pentosane polysulfate [264]. The women underwent cystoscopy and were treated with injections of the peptide BPC-157 (total of 10 mg) around the area of inflammation of the bladder during a single procedure. A Global Response Assessment questionnaire was given to all of the subjects to assess the efficacy of BPC-157, and all 12 patients scored a 5/5 on the Global Response Assessment. Complete resolution of symptoms after one treatment was reported in 10 out of 12 patients, who rated their success at 100%. The remaining 2 out of 12 patients rated their success at 80%, with most symptoms resolved but about 20% of their symptoms lingering. No one dropped out of the study, and no adverse events were reported. This therapy was successful. An improvement in symptoms (urgency and frequency of urination) occurred as early as 2 weeks after the procedure. None of the participants experienced symptoms of fever, skin rash, nausea or vomiting, irritative urinary symptoms (urgency or frequency), or dyspareunia. No postprocedural complications of hematuria or acute cystitis were reported. Illustratively, a cystoscopic image of the same area of the same bladder 6 weeks after intravesical administration of 10 mg BPC-157 demonstrated that initially severe interstitial cystitis with hyperemia, hypervascularity, and hypertrophy of the detrusor muscles showed resolution of the hypervascularity, hyperemia, and hypertrophy of the detrusor muscle [264].

It is noteworthy that these findings are similar to those of several rat studies, evidencing in the case of the BPC 157 therapy a consistent transfer of animal data [180,265,266,267,268].

Thus, the scarce human studies performed so far, regardless of all of the limitations in some of them (i.e., they lacked a large sample size, ethnic variation, and a sham control group), encompassed a wide range of investigations (i.e., ulcerative colitis [65], knee pain [263], and interstitial cystitis [264]). Together, these clinical human data can join the large range of beneficial effects of the BPC 157 therapy indicated by the animal experiments.

In addition to this conclusion, quite a large amount of anecdotal evidence exists regarding the uncontrolled use of BPC 157 preparation for various indications, with regularly positive outcomes, although unconfirmed.

Furthermore, there is an interesting notation provided by Lee and Burgess [261]. In 2024, a task force consisting of physicians, pharmacists, and attorneys conducted an unpublished survey to preserve peptides ahead of the Pharmacy Compounding Advisory Committee (PCAC) meetings on October 29 and 4 December 2024 [269,270]. The survey examined the number of peptide prescriptions dispensed by compounding pharmacies between 2018 and 2024 and whether any side effects had been reported. During this period, 503A compounding pharmacies filled over 500,000 prescriptions for BPC-157 [269,270]. No side effects of BPC-157 were reported by patients to these pharmacies. Additionally, anecdotal reports from physicians indicated no observed side effects following BPC-157 administration via injection or intravenous infusion.

## 6. Transfer of Animal Data and Reliability of Results

Commonly, as pointed out [1], as a general problem of reliability of results and generalizability of findings that could also be important for BPC 157 research, basic studies can have the uncertain translatability of animal data to human contexts. In particular, besides uncertain translatability, many preclinical studies suffer from the lack of comparative evaluations between different delivery methods in one pathological model (i.e., oral vs. intraperitoneal and others). Contrarily, BPC 157 research presents a notable divergence from conventional pharmacological study limitations, particularly in addressing the challenges related to translational validity and administration routes. Notably, studies have systematically evaluated the compound’s efficacy following oral, intraperitoneal, and intragastric administration, often within the same pathological model [3,4,5,6,7,8,9,10,11,12,13,14,16,17,18,19,20,21,22,23,24,27,28,29,30,31,32,34,37,39,40]. This approach enhances the robustness of the data and contributes to a more comprehensive pharmacodynamic profile.

Note, animal-to-human translation is a complex issue (for review see i.e., [271,272,273,274], even when the wide range of translational success rates is consistently evidenced [273]. For example, the percentage of overall correct predictions reported by Litchfield is 74% when both rats and dogs are considered [274]. These data were used to calculate specificity (72%), sensitivity (76%), positive predictive value (68%), and negative predictive value (79%) [274]. While the history of the widely used animal model is always interesting [7], some of the models used in our studies were directly derived from human data. MPTP for Parkinson’s disease has been known since 1982 [224]. Ketamine as a model of schizophrenia (as acute ketamine administration was associated with schizophrenia-like or psychotomimetic symptoms with large effect sizes, an increase in positive and negative symptoms [275,276]) might escape from the extraordinary complexity of extrapolation from animal models of mental disorders in general [277]. Probably not only in theory, these models well used can exemplify the accuracy of other findings as well.

Furthermore, as mentioned, a lack of comparison of different methods of drug administration in one model that may be a general problem [1], is not a problem in BPC 157 animal studies. There were plenty of comparisons of different methods of drug administration in one model (i.e., nine different comparisons) as follows:

i. Intraperitoneal vs. per oral [99,118,140,142,155,169,170,171,194,203,227,229,233,238,239,241,242,265,266,267,278,279,280,281,282,283,284,285,286];
ii. A thin layer of the cream at the site of injury vs. intraperitoneal [82,83,87,287];
iii. Topical at the injured nerve, intraperitoneal, intragastric [288];
iv. Intraperitoneal vs. intragastric vs. intrarectal [289,290];
v. Intramuscular vs. percutaneous into the bone defect [291];
vi. Intramuscular vs. intragastric [292];
vii. Topical application at the brain vs. intraperitoneal vs. intragastric [133];
viii. Eye drops vs. intraperitoneal vs. per oral [113];
ix. Eye drops vs. intraperitoneal [111].

Thus, given a large range of comparisons of different methods of drug administration in one model, along with the used animal models, the obtained results could be regarded as very reliable. The similar beneficial results obtained after different application routes are consistent with the high applicability of the agent whatever route of the application.

## 7. Proving of the Findings’ Relevance by the Extent of Background Concept Development and Realization, and the Extent of the Research

Regularly, the search for new drugs and new developments follows the previous paths in the corresponding concepts and realization of the intended tools. Still, there is a growing recognition of the need for innovative approaches that break from traditional paths in the established concepts to address the resolution of more complex biomedical challenges. Novel drugs intend to avoid or minimize, or at best, intentionally resolve gaps and pitfalls already indicated during the concept’s establishment and previous attempts to realize intended tools.

Therefore, the credibility of all findings in the BPC 157 therapy story [3,4,5,6,7,8,9,10,11,12,13,14,16,17,18,19,20,21,22,23,24,27,28,29,30,31,32,34,37,39,40] is tightly dependent on the relevance and general accuracy of the grand concepts of Robert and Szabo cytoprotection/organoprotection [41,42,43,44,45,46,47,48,49]. The concepts hold the development and counteraction of stomach cell necrosis are relevant for all other organs [39,40,41,42,43,44,45,46,47]. In the same way, in the stomach, endothelial lesions, Virchow triad circumstances, development, and counteraction are relevant for all other organs. Likewise, the credibility of all findings in the BPC 157 therapy also depends on the antecedent Hans Selye’s stress concept, as such [59,60,61] (note, Robert and Szabo concepts [41,42,43,44,45,46,47,48,49] mirror Hans Selye’s stress concept) [59,60,61]. Furthermore, the credibility of the findings should be regarding the possibility that the final endpoint of these concepts (cytoprotection → organoprotection [41,42,43,44,45,46,47,48,49], reestablished body homeostasis [59,60,61]), “response as such” as claimed by both Robert and Selye [41,59], could ever be realized by the pharmacologic agent. Furthermore, Robert and Selye [41,59] “response as such” requires a particular dual activity considering a variety of diverse, even opposite, noxious agents and events. Ultimately, despite the most extensive research encompassing all major organ systems, and beneficial effects, the full spectrum of beneficial effects necessary to fully validate conceptual tools—ranging from cytoprotection to organoprotection and the reestablishment of systemic homeostasis—remains elusive. Besides, these met with a common requirement for adverse effects (any of the therapeutic interventions have side effects). Contrarily, there is that such general therapy involving all organs, together and in particular, should not produce any harm on the other side.

Thus, while further human trials are essential, it remains to be seen to what extent BPC 157—a compound with primarily preclinical support, a wide range of pleiotropic effects, and no observed LD₁ in toxicology studies [3,4,5,6,7,8,9,10,11,12,13,14,16,17,18,19,20,21,22,23,24,27,28,29,30,31,32,34,37,39,40]—can truly achieve such outcomes through pharmacological means. Note, the similar beneficial results of BPC 157 therapy obtained after different application routes are consistent with the high applicability of the agent, whatever route of the application [3,4,5,6,7,8,9,10,11,12,13,14,16,17,18,19,20,21,22,23,24,27,28,29,30,31,32,34,37,39,40] overwhelms limited applicability and activity to prophylactic application only of standard cytoprotective agents [41,42,43,44,45,46,47,48,49]. This clearly shows avoiding pitfalls already indicated during the concept establishment and attempts to realize concept’s tools. In addition, the prompt activation of the collateral pathways by BPC 157 therapy appeared as a new essential point [129,130,131,132,133,134,135,136,137,138,139,140,141,142,168].

On the other hand, the concept and related agents met a fiery end after the concept had failed, and supposed agents and/or mediators were unable to realize theory in praxis. As an illustration, as pointed out in 1975 by J. W. Mason, Selye acknowledged a major weak point in his stress theory, the failure to identify experimentally such a first mediator [293,294]. The search for such physiological first mediators of stress responses, however, has continued to remain largely unproductive up to that time [293,294]. And finally, as pointed out later in the 1980s [295], Selye’s stress (general adaptation syndrome, derangement of which for many stress-induced diseases) has been largely discharged, and replaced by new, humbler ideas, but more precise in nature [295]. However, as later evidenced, BPC 157 therapy resists and counteracts opposite circumstances, i.e., hypotension vs. hypertension, hyperthermia vs. hypothermia [3,4,5,6,7,8,9,10,11,12,13,14,16,17,18,19,20,21,22,23,24,27,28,29,30,31,32,34,37,39,40], as an essential requirement envisaged by Mason [293,294] for the resolving role in general adaptive syndrome.

Given that the NO-system has a general significance [187,188,189], BPC 157/NO-system studies could be an illustration of the extent of the research. All of the studies were carried out with a stable range of NO-agents (L-NAME (5 mg/kg) and L-arginine (100 mg/kg) alone and/or together, and BPC 157 10 µg/kg [11,29]. More than 80 distinctive targets were included in the search. NO-system “body mapping” was done based on the many distinctive NO-system responses shown by distinctive NO-agents relations [11,29]. Demonstration was that BPC 157 indeed counteracted diverse adverse effects of L-NAME, diverse adverse effects of L-arginine, and the consequences of combined application of L-NAME and L-arginine [11,29]. However, it remains how this dual regulation is molecularly/mechanically achieved. Note, quite immediately, Fourier transform infrared spectroscopy reveals molecular changes in blood vessels of rats treated with pentadecapeptide BPC 157 [143]. Also, the evidence demonstrates modulatory effects of BPC 157 on vasomotor tone and the activation of the Src-Caveolin-1-endothelial NOS pathway [93]. BPC 157 not only increased VEGFR2 expression in vascular endothelial cells several hours after treatment but also rapidly induced VEGFR2 internalization within minutes, subsequently activating the phosphorylation of VEGFR2, Akt, and the eNOS signaling pathway, independent of known ligands or shear stress [93].

## 8. Final Remarks and Conclusions

**Figure 4:**
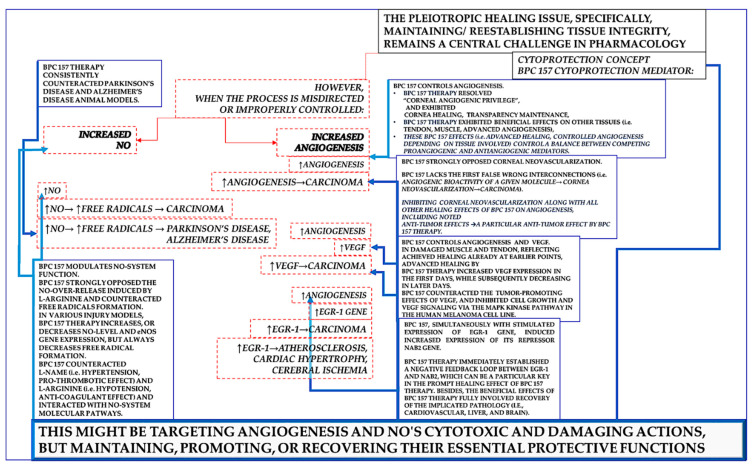
Summary of BPC 157, a cytoprotection mediator (small black box), pleiotropic beneficial effects anchored to its resolving effects on increased angiogenesis, increased VEGF, increased egr-1 gene, increased NO, or eNOS stimulation, and increased free radical formation. Commonly, the pleiotropic issue of healing (i.e., specifically, maintaining/reestablishing tissue integrity) remains a central challenge in pharmacology (black box), particularly when the process is misdirected or not properly controlled (red lines, arrows, and boxes). The specific issue could be a strong increasing effect on particular targets, increasing angiogenesis, increased VEGF, increased egr-1 gene, increased NO or eNOS stimulation, and increased free radical formation (red boxes to indicate adverse outcomes, dashed to indicate counteraction by BPC 157 therapy). Therefore, all of these items (blue boxes), properly accommodated by BPC 157 therapy (i.e., cytoprotection concept implementation, regulation of the increasing angiogenesis, increased VEGF, increased egr-1 gene, increased NO, or eNOS stimulation, and counteraction of the increased free radical formation), might be a key to resolving beneficial action (blue lines and arrows). This might be the updated concept of cytoprotection as, long ago, innate epithelial and endothelial cell protection was postulated in the stomach. This might include targeting angiogenesis and NO’s cytotoxic and damaging actions but maintaining, promoting, or recovering their essential protective functions. Thus, this would occur as a highly controlling beneficial action, activating an effect ascribed to BPC 157 therapy’s effect depending on the disturbed circumstances. Manifest risks of unanticipated adverse effects due to pleiotropic effects and interaction with multiple systems did not occur. BPC 157 therapy’s effects are safe concerning the cure of each organ involved, including the brain, heart, lung, liver, kidney, and gastrointestinal tract, and they do not produce any adverse effect on account of its beneficial effects, along with other findings, i.e., so far no reported adverse effects in basic research, LD1 not achieved, no adverse effects in clinical trials.

With all of these caveats, specifically, in wound healing and general healing capabilities, as reviewed [3,4,5,6,7,8,9,10,11,12,13,14,16,17,18,19,20,21,22,23,24,27,28,29,30,31,32,34,37,39,40], as a cytoprotective agent and native cytoprotection mediator, BPC 157 controls angiogenesis and the NO-system healing functions as a whole. There, the pleiotropic beneficial effect, along with cytoprotection concept implementation, regulation of the increasing angiogenesis, increased VEGF, increased egr-1 gene, increased NO, or eNOS stimulation, and counteraction of the increased free radical formation, occur through a network of closely interconnected pathways presented in this review (see Section 1, Section 2 and Section 3). This might be targeting angiogenesis and NO’s cytotoxic and damaging actions but maintaining, promoting, or recovering their essential protective functions. Thus, this would occur as a highly controlling beneficial action, activating an effect ascribed to BPC 157’s therapeutic effect, depending on the disturbed circumstances. Manifest risks of unanticipated adverse effects due to pleiotropic effects and interaction with multiple systems did not occur. BPC 157’s therapeutic effects are safe concerning the cure of each organ involved, including the brain, heart, lung, liver, kidney, and gastrointestinal tract, and it does not produce any adverse effect on account of its beneficial effects, along with other findings, i.e., so far, no reported adverse effects in basic research, LD1 not achieved, and no adverse effects in clinical trials. As final proof of the concept, it counteracts the pathological presentation of neurodegenerative diseases in acknowledged animal models (i.e., Parkinson’s disease and Alzheimer’s disease) and presents prominent anti-tumor potential in vivo and in vitro (Figure 4).

The pleiotropic issue of healing (i.e., specifically, maintaining/reestablishing tissue integrity) is a central and not completely understood problem in pharmacology, which many concepts attempt to approach. The specific issue could be a strong effect on increasing angiogenesis, increased VEGF, increased egr-1 gene, increased NO or eNOS stimulation, and increased free radical formation, interaction with multiple systems, and unescapable risks of unanticipated adverse effects due to pleiotropic effects. To avoid and manage these problems, one approach is Robert and Szabo’s concept of cytoprotection, which holds innate cell (epithelial (Robert) [41,42,43] endothelial (Szabo) [43,44,45,46,47]) integrity and protection/maintenance/reestablishment in the stomach to be translated to other organ therapy (cytoprotection → organoprotection) [48,49] via the cytoprotection agent’s effect.

Being a cytoprotection mediator native and stable in human gastric juice, suggests its easy applicability, including the per oral route, and strong distinction from the standard angiogenic growth factors, whose effects regularly need carrier addition [3,4,5,6,7,8,9,10,11,12,13,14,16,17,18,19,20,21,22,23,24,27,28,29,30,31,32,34,37,39,40]. Furthermore, BPC 157 therapy in severe conditions involving multiple organs and vascular failure in counteracting occlusion/occlusion-like syndrome as a whole offers illustrative examples of how the risks associated with this complexity can be managed or resolved. BPC 157 therapy’s effects are safe concerning the cure of each organ involved, including the brain, heart, lung, liver, kidney, and gastrointestinal tract, and it does not produce any adverse effect on account of its beneficial effects [129,130,131,132,133,134,135,136,137,138,139,140,141,142,168], along with other findings, i.e., so far no reported adverse effects in basic research, LD1 not achieved, no adverse effects in clinical trials [3,4,5,6,7,8,9,10,11,12,13,14,16,17,18,19,20,21,22,23,24,27,28,29,30,31,32,34,37,39,40]. Thus, regardless of some disputes [1], we can defend BPC 157 therapy for targeting angiogenesis and NO’s cytotoxic and damaging actions but maintaining, promoting, or recovering their essential protective functions [3,4,5,6,7,8,9,10,11,12,13,14,16,17,18,19,20,21,22,23,24,27,28,29,30,31,32,34,37,39,40].

Finally, targeting angiogenesis and NO’s cytotoxic and damaging actions but maintaining, promoting, or recovering their essential protective functions [3,4,5,6,7,8,9,10,11,12,13,14,16,17,18,19,20,21,22,23,24,27,28,29,30,31,32,34,37,39,40] could indicate cytoprotective evidence that aligns with BPC 157 as a neurotransmitter, as previously envisaged in the implementation of the cytoprotection effects [6,9] (i.e., a cytoprotection mediator holds a response specifically related to preventing or recovering damage as such). In this, it might be the question of whether the cytoprotection concept is valid or not. Nevertheless, although BPC 157 lacks general standard neurotransmitter criteria, a network of interconnected evidence has demonstrated that BPC 157 therapy counteracts, in addition to NO-system disturbances, also dopamine, serotonin, glutamate, GABA, adrenalin/noradrenalin, and acetylcholine disturbances, whether specifically related to their receptors, including both blockade and over-activity, destruction, depletion, tolerance, and sensitization, and there is also channel disturbance counteraction [6,9]. Note that some believe that these should be described as a “transmitter modulator” (personal communication). Furthermore, as previously pointed out, the close BPC 157/NO-system relationship with the gasotransmitters crossing the cell membrane and acting directly on molecules inside of the cell may suggest particular interactions with receptors on the plasma membrane of their target cells [6,9]. On the other hand, BPC 157, a stable pentadecapeptide native and stable in human gastric juice, can be released into circulation as a cytoprotective mediator and sent to distant organs [3,4,5,6,7,8,9,10,11,12,13,14,16,17,18,19,20,21,22,23,24,27,28,29,30,31,32,34,37,39,40]. Indeed, through in situ hybridization and immunostaining, BPC 157 was found in humans, in both adult and fetal tissues, gastrointestinal mucosa, lung bronchial epithelium, the epidermal layer of the skin, and kidney glomeruli [21], and therefore may have a regulatory role. Possibly, the similar beneficial effects in other species (i.e., birds [296] and insects [297,298,299]) may suggest that BPC 157 may also have an extended regulatory physiologic role in bodily functions. Finally, BPC 157 therapy has been tested in reliable experiments by taking a comparison of several application routes in the same model and obtaining congruence between various application routes, as requested proof (see Section 6).

In practice, if the cytoprotection concept is valid as a general concept (cytoprotection → organoprotection) [41,42,43,44,45,46,47,48,49] and the NO-system has general significance [189,190,191], they can serve each other’s functions accordingly via cytoprotection agent modulatory potential on NO-blockade/NO-over-activity/NO-immobilization. Thus, with such a background (i.e., useful modulation/control of the increased angiogenesis, elevated VEGF levels, upregulation of the egr-1 gene, enhanced NO and eNOS stimulation, and counteraction of increased free radical formation), stable gastric pentadecapeptide BPC 157 is working as a cytoprotective agent (and, therefore, with a highly postulated safety profile) (and not as a “panacea” effective for all conditions). This explains the particular aspects of the stable gastric pentadecapeptide BPC 157’s pleiotropic beneficial activity as a part of its cytoprotective (organoprotective) activity and its consistent effectiveness using various application routes. As reviewed before [3,4,5,6,7,8,9,10,11,12,13,14,16,17,18,19,20,21,22,23,24,27,28,29,30,31,32,34,37,39,40], BPC 157’s µg-ng dose range applied alone, without carrier addition, and its application including via the per oral route, clinical evidence, and toxicology without LD1 can ascertain its practical applicability.

## Figures and Tables

**Table 1:** The relevance of stable gastric pentadecapeptide BPC 157 based on the multifunctionality and possible medical application of stable gastric pentadecapeptide BPC 157, as seen in the presented reviews [1,2,3,4,5,6,7,8,9,10,11,12,13,14,15,16,17,18,19,20,21,22,23,24,25,26,27,28,29,30,31,32,33,34,35,36,37,38,39,40].

Multifunctionality and Possible Medical Application of Stable Gastric Pentadecapeptide BPC 157, as Seen in the Presented Reviews [1,2,3,4,5,6,7,8,9,10,11,12,13,14,15,16,17,18,19,20,21,22,23,24,25,26,27,28,29,30,31,32,33,34,35,36,37,38,39,40]	
Józwiak et al. Multifunctionality and possible medical application of the BPC 157 peptide-literature and patent review. doi: 10.3390/ph18020185. PMID: 40005999 [1]	*Pharmaceuticals* (Basel). 2025;18(2):185.
DeFoor and Dekker. Injectable therapeutic peptides—an adjunct to regenerative medicine and sports performance? [2]	*Arthroscopy*. 2025 Feb;41(2):150–152.
Grubisic et al. Stable gastric pentadecapeptide BPC 157 as a therapy of severe electrolyte disturbances in rats. [3]	*Curr Neuropharmacol*. 2025 Jan 24. doi: 10.2174/011570159X349612241205065330. Online ahead of print. PMID: 39865815
Sikiric et al. New studies with stable gastric pentadecapeptide protecting gastrointestinal tract. Significance of counteraction of vascular and multiorgan failure of occlusion/occlusion-like syndrome in cytoprotection/organoprotection. [4]	*Inflammopharmacology*. 2024 Oct;32(5):3119–3161.
Bajramagic et al. Stable gastric pentadecapeptide BPC 157 and intestinal anastomoses therapy in rats—a review. [5]	*Pharmaceuticals* (Basel). 2024 Aug 17;17(8):1081.
Sikiric et al. The stable gastric pentadecapeptide BPC 157 pleiotropic beneficial activity and possible relations with neurotransmitter activity. [6]	*Pharmaceuticals* (Basel). 2024 Apr 3;17(4):461.
Sikiric et al. From Selye’s and Szabo’s cysteamine-duodenal ulcer in rats to dopamine in the stomach: Therapy significance and possibilities. [7]	*Pharmaceuticals* (Basel). 2023 Dec 7;16(12):1699.
Sikiric et al. Stable gastric pentadecapeptide BPC 157—possible novel therapy of glaucoma and other ocular conditions. [8]	*Pharmaceuticals* (Basel). 2023 Jul 24;16(7):1052.
Sikiric et al. Stable gastric pentadecapeptide BPC 157 may recover brain-gut axis and gut-brain axis function. [9]	*Pharmaceuticals* (Basel). 2023 Apr 30;16(5):676.
Sikiric et al. Stable gastric pentadecapeptide BPC 157: Prompt particular activation of collateral pathways. [10]	*Curr Med Chem*. 2023;30(13):1568–1573.
Sikiric; et al. Stable gastric pentadecapeptide BPC 157 and NO-system [11]	In: *Nitric Oxide: From Research to Therapeutics, Advances in Biochemistry in Health and Disease 22*; Ray, A., Gulati, K., Eds.; Springer Nature: Cham, Switzerland, 2023; pp. 349–375.
Staresinic et al. Stable gastric pentadecapeptide BPC 157 and striated, smooth, and heart muscle. [12]	*Biomedicines*. 2022 Dec 12;10(12):3221.
Sikiric et al. Stable gastric pentadecapeptide BPC 157 as useful cytoprotective peptide therapy in the heart disturbances, myocardial infarction, heart failure, pulmonary hypertension, arrhythmias, and thrombosis presentation. [13]	*Biomedicines*. 2022 Oct 25;10(11):2696.
Vukojevic et al. Pentadecapeptide BPC 157 and the central nervous system. [14]	*Neural Regen Res*. 2022 Mar;17(3):482–487.
Deek. BPC 157 as potential treatment for COVID-19. [15]	*Med Hypotheses*. 2021 Nov 9;158:110736.
Seiwerth et al. Stable gastric pentadecapeptide BPC 157 and wound healing. [16]	*Front Pharmacol*. 2021 Jun 29;12:627533.
Sikiric et al. Stable gastric pentadecapeptide BPC 157, Robert’s stomach cytoprotection/adaptive cytoprotection/organoprotection, and Selye’s stress coping response: Progress, achievements, and the future. [17]	*Gut Liver*. 2020 Mar 15;14(2):153–167.
Park et al. BPC 157 rescued NSAID-cytotoxicity via stabilizing intestinal permeability and enhancing cytoprotection. [18]	*Curr Pharm Des*. 2020;26(25):2971–2981.
Sikiric et al. Fistulas healing. Stable gastric pentadecapeptide BPC 157 therapy. [19]	*Curr Pharm Des*. 2020;26(25):2991–3000.
Gwyer et al. Gastric pentadecapeptide body protection compound BPC 157 and its role in accelerating musculoskeletal soft tissue healing. [20]	*Cell Tissue Res*. 2019 Aug;377(2):153–159.
Seiwerth et al. BPC 157 and standard angiogenic growth factors. Gastrointestinal tract healing, lessons from tendon, ligament, muscle and bone healing. [21]	*Curr Pharm Des*. 2018;24(18):1972–1989.
Kang et al. BPC157 as potential agent rescuing from cancer cachexia. [22]	*Curr Pharm Des*. 2018;24(18):1947–1956.
Sikiric et al. Novel cytoprotective mediator, stable gastric pentadecapeptide BPC 157. Vascular recruitment and gastrointestinal tract healing. [23]	*Curr Pharm Des*. 2018;24(18):1990–2001.
Sikiric et al. Stress in gastrointestinal tract and stable gastric pentadecapeptide BPC 157. Finally, do we have a solution? [24]	*Curr Pharm Des*. 2017;23(27):4012–4028.
Szabo et al. “Stress” is 80 years old: From Hans Selye original paper in 1936 to recent advances in GI ulceration. [25]	*Curr Pharm Des*. 2017;23(27):4029–4041.
Gyires and Feher A. Stress, neuropeptides and gastric mucosa. [26]	*Curr Pharm Des*. 2017;23(27):3928–3940.
Sikiric et al. Brain-gut axis and pentadecapeptide BPC 157: Theoretical and practical implications. [27]	*Curr Neuropharmacol*. 2016;14(8):857–865.
Seiwerth et al. BPC 157 and blood vessels. [28]	*Curr Pharm Des*. 2014;20(7):1121–5.
Sikiric et al. Stable gastric pentadecapeptide BPC 157-NO-system relation. [29]	*Curr Pharm Des*. 2014;20(7):1126–35
Sikiric et al. Toxicity by NSAIDs. Counteraction by stable gastric pentadecapeptide BPC 157. [30]	*Curr Pharm Des*. 2013;19(1):76–83.
Sikiric et al. Focus on ulcerative colitis: stable gastric pentadecapeptide BPC 157. [31]	*Curr Med Chem*. 2012;19(1):126–32.
Sikiric et al. Stable gastric pentadecapeptide BPC 157: novel therapy in gastrointestinal tract. [32]	*Curr Pharm Des*. 2011;17(16):1612–32.
Mózsik et al. Approaches to gastrointestinal cytoprotection: from isolated cells, via animal experiments to healthy human subjects and patients with different gastrointestinal disorders. [33]	*Curr. Pharm. Des*. 2011,17(16),1556–1572.
Sikiric et al. Revised Robert’s cytoprotection and adaptive cytoprotection and stable gastric pentadecapeptide BPC 157. Possible significance and implications for novel mediator. [34]	*Curr Pharm Des*. 2010;16(10):1224–34.
Mózsik. Gastric cytoprotection 30 years after its discovery by Andre Robert: a personal perspective. [35]	*Inflammopharmacology* 2010,18(5),209–221.
Mózsik et al. Gastrointestinal cytoprotection: from basic science to clinical perspectives. [36]	*Inflammopharmacology* 2007,15(2),49–60.
Sikiric et al. Stable gastric pentadecapeptide BPC 157 in trials for inflammatory bowel disease (PL-10, PLD-116, PL 14736, Pliva, Croatia). Full and distended stomach, and vascular response. [37]	*Inflammopharmacology*. 2006 Dec;14(5–6):214–21.
Wood. The first Nobel prize for integrated systems physiology: Ivan Petrovich Pavlov, 1904. [38]	*Physiology*, 2004,19(6),326–330.
Sikiric The pharmacological properties of the novel peptide BPC 157 (PL-10). [39]	*Inflammopharmacology*. 1999;7(1):1–14.
Sikiric et al. A new gastric juice peptide, BPC. An overview of the stomach-stress-organoprotection hypothesis and beneficial effects of BPC. [40]	*J Physiol Paris*. 1993;87(5):313–27

**Table 2:** Summary of the effects of controlled angiogenesis, counteracted corneal neovascularization [76], advanced angiogenesis in the healing of transected Achilles tendon [90], and counteracted pathologic angiogenesis (bile duct ligation-induced liver cirrhosis, 8 weeks) in liver cirrhosis healing through BPC 157 therapy [118].

References	Organ System	Dose and Protocol	Model	Key Findings
Masnec et al. Perforating corneal injury in rat and pentadecapeptide BPC 157. *Exp. Eye Res.* 2015, *136*, 9–15. [76]	Cornea	After injury induction, BPC 157 therapy successfully closed perforating corneal incisions in rats and rapidly restored corneal transparency. This effect is quite consistent given the regimens used, i.e., 2 pg/mL, 2 ng/mL, and 2 µg/mL distilled water, two eye drops/left rat eye immediately after injury induction, and then every 8 h up to 120 h.	Perforating corneal injury	BPC 157 therapy cured severe corneal lesions and maintained corneal transparency. All controls developed new vessels that grew from the limbus to the penetrated area and had no transparency. Contrarily, BPC 157-treated rats generally had no new vessels, and those that did form in the limbus did not make contact with the penetrated area.
Staresinic et al. Gastric pentadecapeptide BPC 157 accelerates healing of transected rat Achilles tendon and in vitro stimulates tendocytes growth. *J. Orthop. Res*. 2003, *21(6)*, 976–983. [90]	Achilles tendon	Agents (/kg b.w., i.p., once time daily) (BPC 157 (dissolved in saline, with no carrier addition) (10 microg, 10 ng or 10 pg) or saline (5.0 mL)) were firstly applied 30 min after surgery, with the last application 24 h before autopsy.	Transected Achilles tendon	Pentadecapeptide BPC 157 fully improves recovery: (i) biomechanically, increased load of failure, load of failure per area, and Young’s modulus of elasticity; (ii) functionally, significantly higher AFI values; (iii) microscopically, more mononuclears and less granulocytes, superior formation of fibroblasts, reticulin, and collagen; (iv) macroscopically, smaller size and depth of tendon defect, and, subsequently, the reestablishment of full tendon integrity. Unlike the damaged cornea [74,75,76,77], in the healing of transected Achilles tendon already at postoperative day 4, BPC 157-treated rats have large fields of dense mature collagen, illustrating well the consistent organ-specific healing effect’s cellularity, and well-formed capillaries and small vessels [88], while control rats exhibit only some young capillaries.
Sever et al. Stable gastric pentadecapeptide BPC 157 in the therapy of the rats with bile duct ligation. *Eur. J. Pharmacol.* 2019, *847*, 130–142. [118]	Liver	In bile duct occluded rats, in an 8-week study, BPC 157 (10 µg/kg, 10 ng/kg) was given continuously (intraperitoneally once a day or perorally (continuously in drinking water)) or only once as a direct bath application.	Bile duct ligation- induced liver cirrhosis	Liver weight was not increased, and ascites was eliminated. Microscopy presentation documented the smaller intensity of architectural changes (fibrosis and cirrhosis); lower necroinflammatory score; smaller alpha-smooth muscle actin (α-SMA) distribution; and smaller Ki-67 distribution. Smaller were serum enzymes and bilirubin values. Normalized were MDA- and NO-levels in the liver, next to Western blot of NOS2 and NOS3 in the liver tissue and decreased IL-6, TNF-α, and IL-1β levels in liver tissues. Annihilation of portal hypertension consistently occurred. Despite bile duct ligation, portal pressure did not develop. With late application of BPC 157 therapy in bile duct ligated rats with already advanced liver cirrhosis, portal hypertension disappeared and did not reappear.

**Table 3:** Distinct NO-response patterns. Utilizing a triple-application approach involving L-NAME, L-arginine, and their combination [11], this table summarizes over 80 distinctive targets and numerous variations in NO-system responses at those sites. The analysis reveals a variety of characteristic NO-agent response patterns, categorized as follows: (i) L-NAME responsive/L-arginine responsive (L-NAME R, L-arginine R); L-NAME responsive/L-arginine non-responsive (L-NAME R, L-arginine NR); L-NAME non-responsive/L-arginine responsive (L-NAME NR, L-arginine R). (ii) These response relationships were further classified based on interaction types: *Opposite*: L-NAME and L-arginine exert opposing effects; *Parallel*: L-NAME and L-arginine exert similar effects. (iii) NO-*specific*: L-NAME and L-arginine counteract each other’s effects, indicative of specific NO-mediated mechanisms. NO-*non-specific*: L-NAME and L-arginine do not counteract each other’s effects, suggesting mechanisms independent of specific NO pathways. (iv) NO-*not relevant*: L-NAME non-responsive/L-arginine non-responsive (L-NAME NR, L-arginine NR). Notably, this triple-application strategy provides a more comprehensive evaluation compared to conventional studies using only a single NO agent (typically, L-NAME) (for a review, see [11]). In the table, applied models are denoted in *italic*, while targets are indicated in regular font.

*Response*	*Target*
L-NAME R, L-arginine R *Opposite*, *specific*	*Perforated cecum*: vessel presentation, bleeding; *Cyclophosphamide*: hemorrhagic cystitis; *short bowel*: liver lesions, brain lesions; *Celecoxib*: gastric lesions, liver lesions; *esophagogastric anastomosis*: anastomosis strength, “esophageal sphincter” function, pyloric sphincter function; *tail amputation*: bleeding; *Warfarin*: bleeding; *duodenocutaneous fistula*: duodenal defect, skin defect, fistula, lethality; *esophagocutaneous fistula*: esophageal defect, skin defect, esophagocutaneous fistula leaking; *Intra(per)-oral/intragastric strong alcohol in rat*: tongue lesions, gastric lesions, duodenal lesions, lower esophageal pressure; *mortal hyperkalemia*: survival and life-saving potential, arrhythmias, hypertension, lower esophageal sphincter pressure, blood pressure; in vitro *in stomach tissue homogenates*: NO generation; *left colic artery and vein ligation*: arcade vessel; *parietal peritoneum excision with an underlying superficial layer of muscle tissue in rats*: increased adhesion formation; *ketamine-induced*: anhedonia
L-NAME R, L-arginine NR *Opposite*, *specific*	*Short bowel*: gastrointestinal lesions, failed anastomosis healing, intestinal adaptation deterioration; *Cyclophosphamide*: gastric lesions, duodenal lesions; *colocutaneous fistula*: colon defect, skin defect, fistula; *mortal hyperkalemia*: muscular disability
L-NAME NR, L-arginine R *Opposite*, *specific*	*Celecoxib*: brain lesions; *heparin*: bleeding; *Apomorphine, MK-801, haloperidol, methamphetamine*: apomorphine-induced disturbances, MK-801-induced locomotion, stereotyped sniffing, ataxia disturbances, haloperidol-induced catalepsy, methamphetamine-induced disturbances; *mortal hyperkalemia*: pyloric sphincter pressure; *intragastric alcohol*: stomach lesions; *ketamine-induced*: cognitive dysfunction
L-NAME R, L-arginine R *Opposite*, *non-specific*	*Esophagogastric anastomosis*: esophagitis lesions, gastric lesions, lethality; *esophagocutaneous fistula*: lower esophageal sphincter pressure; *ketamine-induced*: social withdrawal
L-NAME R, L-arginine NR *Opposite*, *non-specific*	*Thiopental*: anesthesia
L-NAME NR, L-arginine NR *Not relevant*	*Perforated cecum*: cecum lesion; *Cyclophosphamide*: leak point pressure; *tail amputation*: APTT-, TT- values; *heparin*: APTT-, TT-values; *Warfarin*: thrombocytopenia, PT-values; *intra(per)-oral/intragastric strong alcohol in rat*: pyloric sphincter pressure; *mortal hyperkalemia*: serum electrolyte values; *superior anterior pancreaticoduodenal vein ligation*: vessels
L-NAME R, L-arginine R¸ *Parallel*, *specific*	*Superior anterior pancreaticoduodenal vein ligation*: duodenal lesions; *hypermagnesemia*: muscle weakness, muscle lesions, brain lesions, hypermagnesemia, hyperkalemia; *tail amputation*: thrombocytopenia; *heparin*: thrombocytopenia; *lidocaine*: lidocaine-induced local anesthesia via intraplantar application, lidocaine-induced axillary block, lidocaine-induced spinal (L4-L5) intrathecal block; *left colic artery and vein ligation*: colon lesions; *parietal peritoneum excision with an underlying superficial layer of muscle tissue in rats*: failed vasculature; *ketamine-induced*: anxiety
L-NAME R, L-arginine R *Parallel, non-specific*	*Normal pupil*: miosis; *atropine-induced*: mydriasis; *dopamine antagonists*: lower esophageal sphincter pressure, pyloric sphincter pressure; *amphetamine*: amphetamine-induced disturbances; *acute alcohol intoxication*: behavior, temperature, alcohol level in blood; *alcohol*: withdrawal; *broiler chicken*: pulmonary hypertension syndrome

**Table 4:** Relevant pharmacokinetic parameters from plasma levels (noncompartmental analysis).

Parameter	Unit	Males	Females	Males +Females
Time of maximum concentration (tmax)	h	8	3	3
Maximum concentration (Cmax)	µg eq./mL	10.36	10.90	10.53
Area under the curve (AUC)	h·µg eq./mL	988	1058	1023
Half-life (t1/2)	h	66	69	68
Mean residence time (MRT)	h	91	94	93
Index of bioavailability (F)	-	1.1	1.2	1.2

The index of bioavailability (F) was calculated using the AUC data obtained in an intravenous pharmacokinetics study with the same test article (RBM Exp. No. 950509). Bioavailability of 110–120% was calculated, which indicates complete absorption of the test article from the gastrointestinal tract (within the 80–125% confidence limits for equivalency). The blood/plasma ratios of radioactivity were near unity in all samples, indicating an equivalent distribution between plasma and blood cells.
